# Unraveling the Optimal Cerium Content for Boosting the Photoresponse Activity of Mixed-Metal Zr/Ce-Based Metal–Organic Frameworks through a Photodynamic and Photocurrent Correlation: Implications on Water Splitting Efficiency

**DOI:** 10.1021/acsami.3c08062

**Published:** 2023-07-21

**Authors:** Arghyadeep Bhattacharyya, Mario Gutiérrez, Boiko Cohen, Horatiu Szalad, Josep Albero, Hermenegildo Garcia, Abderrazzak Douhal

**Affiliations:** † Departamento de Química Física, Facultad de Ciencias Ambientales y Bioquímica, and INAMOL, Universidad de Castilla-La Mancha, Avenida Carlos III, S.N., 45071 Toledo, Spain; ‡ Instituto de Tecnología Química, Consejo Superior de Investigaciones Científicas-Universitat Politècnica de València, Universitat Politecnica de Valencia, Av. De los Naranjos s/n, 46022 Valencia, Spain

**Keywords:** mixed-metal MOFs, time-resolved spectroscopy, LCCT, photocurrent, photoresponse, water splitting

## Abstract

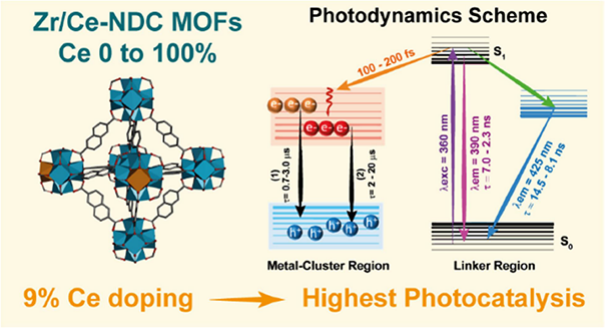

Mixed-metal–organic frameworks (MMOFs) have emerged as promising photocatalyst candidates in multiple reactions. For instance, the doping of Zr-UiO-type MOFs with Ce atoms increases their photoactivity owing to a better overlap between the organic linker and Ce orbitals. However, it is not clear which is the ideal content of Ce to reach the optimal photocatalytic performance. Herein, a series of MMOFs isostructural to UiO-66 and with napthalene-2,6-dicarboxylate (NDC) as a linker were synthesized and characterized. The Ce content was varied from 0 to 100% and their corresponding structural, chemical, photodynamic, and photoresponse properties were investigated. Powder X-ray diffraction shows that when the content of Ce is 12% onward, in addition to the UiO-type structure, a second crystalline structure is cosynthesized (NDC-Ce). Steady-state and femtosecond (fs) to millisecond (ms) spectroscopy studies reveal the existence of two competing processes: a linker excimer formation and an ultrafast ligand-to-cluster charge transfer (LCCT) phenomenon from the organic linker to Zr/Ce metal clusters. The ultrafast (fs-regime) LCCT process leads to the formation of long-lived charge-separated states, which are more efficiently photoproduced when the content of Ce reaches 9%, suggesting that the related material would show the highest photoactivity. Photoaction spectroscopic measurements corroborate that the sample with 9% of Ce exhibits the maximum photocatalytic efficiency, which is reflected in a 20% increment in overall water splitting efficiency compared with the monometallic Zr-based MOF. The current study demonstrates the relationship between the photodynamical properties of the MMOFs and their photocatalytic performance, providing new findings and opening new ways for improving the design of new MOFs with enhanced photocatalytic activities.

## 1 Introduction

Metal–organic frameworks (MOFs), a special class of crystalline porous materials constructed with metals ions or clusters interlinked by organic ligands,^1^ have attracted the attention of a large number of multidisciplinary researchers owing to their potential implementation in forefront technologies and scientific advancements.^2−6^ The chemical versatility of these materials has boosted the fabrication of different MOF structures with bespoke physicochemical properties. Indeed, nowadays it is possible to design photoactive MOFs on demand,^7,8^ which have been used in different fields of photonics spanning from sensing, optoelectronic applications, cell imaging, and photocatalysis.^9−17^ Due to their large surface area,^18^ ordered porous structure,^19^ tunable organic bridging linker/metal clusters,^20^ higher thermal stability,^21^ and better water tolerance than most of other MOFs,^22^ Zr-based MOFs isostructural to the UiO family, have garnered great interest for their potential as photocatalysts in different reactions.^23−27^ However, the UiO-type MOFs are mainly constituted by *d*^0^ metals such as Zr or Hf, whose binding energy is too low, and consequently, the overlap between their orbitals and the π*-orbitals of the organic linkers is very inefficient.^28,29^ This mismatch in the orbital levels hinders an efficient generation of long-lived charge-separated states (CSSs) in the photoexcited MOFs, which are the cornerstone of the photocatalytic reactions, and therefore reduces the photocatalytic activity of MOFs like Zr-UiO or Hf-UiO.^28^ To circumvent this drawback, it has been proposed that doping the Zr clusters with Ce in UiO-isostructural MOFs may enhance the photocatalytic activity as a result of a more efficient ligand-to-cluster charge transfer (LCCT) process upon photoexcitation of the organic linkers.^30−34^ The enhancement of the LCCT event is caused by the low-lying empty 4f orbitals of Ce^4+^ that better overlap with the π*-orbitals of the organic linkers.^32^ Theoretical and experimental reports have demonstrated that a partial substitution of Zr by Ce atoms in UiO MOFs produced better photocatalytic activities compared to the pristine Zr-based MOFs.^35−37^ However, there is no clear guidance to what extent the Zr-UiO MOFs should be doped with Ce for reaching the optimal photocatalytic efficiency.

In parallel, another complimentary approach to boost the photocatalytic activity of UiO-type MOFs is to replace the commonly used benzene dicarboxylate linker in UiO-66 MOFs by the π-electron-rich naphthalene dicarboxylate one.^38,39^ Although the UiO-66-based MOFs have been extensively explored and the connections between the photodynamical events occurring in their excited states and their photocatalytic activities have been unraveled,^30,40,41^ this information is scarce for the corresponding naphthalene-based analogues.

Thus, herein we synthesized a series of mixed-metal Zr/Ce-NDC (NDC = 2,6-napththalene dicarboxylate) MOFs. The percentage of Ce in these MOFs was incremented from 0 to 2, 5, 9, 12, 25, 50, 75, and 100% to unveil which is the most suitable content of Ce for improving the photocatalytic properties of these materials. The structural, chemical, morphological, spectroscopic, and photodynamic properties of all these materials have been characterized. From the structural and chemical characterization, we found that for the MOFs with 12% Ce onward, a secondary crystalline structure, in addition to the UiO crystalline one, was cosynthesized. This secondary phase is attributed to the formation of a monometallic NDC-Ce MOF, which is different from the Ce-UiO-NDC MOF previously reported that exhibits the same diffraction pattern as that of Zr-DUT-52.^42^ We demonstrate that the spectroscopic properties of the MOFs strongly depend on the content of Ce. For instance, the emission spectrum of the MOFs containing 0–9% of Ce consists of a vibrationally resolved band, whose emission intensity clearly diminishes with the content of Ce. On the other hand, the emission spectrum of the MOFs with 12–100% of Ce shows a broad structureless band, which is red-shifted in comparison to the other Zr/Ce-based MOFs. This difference is ascribed to the formation of the NDC-Ce crystalline phase, as revealed by the powder X-ray diffraction (PXRD) results of the MOFs. Similarly, the photodynamic properties of these MOFs are affected by the content of Ce. As previously reported, the photodynamics of the pristine Zr-NDC MOF shows a multiexponential behavior attributed to the emission lifetimes of NDC monomers and excimers along with a shorter component ascribed to the excimer photoformation.^43−46^ The observed trend in the photodynamics of the MOFs studied here is a decrease in the lifetime values with the content of Ce as a consequence of a more efficient LCCT due to the better overlap of the linker and Ce orbitals. In fact, the study of the nonradiative photoprocesses of these MOFs demonstrates the existence of an ultrafast LCCT process, leading to the formation of long-lived (microsecond regime) CSSs. Remarkably, the formation of CSSs is more efficient for the sample containing 9% of Ce, suggesting that this sample should exhibit the highest photocatalytic response. To explore this possibility, we performed transient photocurrent experiments and proved that the MOF with 9% of Ce exhibits the highest photocurrent in this series of MOFs. This result is in agreement with the 20% enhancement on the overall water splitting photocatalytic performance of the MOF containing 9% of Ce when compared with the monometallic Zr-based MOF. However, we observed that a larger amount of Ce induces the formation of another crystalline phase (i.e., NDC-Ce), which results in a decrease of the photocurrent activity of the bulk material.

Our results clearly demonstrate a correlation between the photodynamic properties of a family of mixed-metal Zr/Ce MOFs and their photoresponse. From these data, we provide a guideline of how to unveil the optimal content of Ce that can be doped in a Zr-NDC MOF for reaching the maximum photocatalytic activity. We also prove that steady-state and time-resolved spectroscopies are powerful tools to predict the photocatalytic response of mixed-metal MOFs, and therefore, the results reported in this work might help in the design and fabrication of advanced MOF photocatalysts. Moreover, we also demonstrate that these particular Zr–Ce-mixed MOFs are relevant materials for water splitting, opening up a path for further efficiency improvement by MOF (co)doping with other metals, such as Ti. Hence, the results presented in this work might be of interest for multidisciplinary researchers, including synthetic chemists, spectroscopists, physicists, and engineers.

## 2 Materials and Methods

### 2.1 Materials

2,6-Napththalene dicarboxylic acid (96%), zirconium(IV) chloride (ZrCl_4_, 98%), ammonium cerium(IV) nitrate ((NH_4_)_2_[Ce(NO_3_)_6_], ≥98.5%), glacial acetic acid, dichloromethane (DCM, anhydrous 99.9%), and *N*,*N*-dimethylformamide (DMF, anhydrous 99.8%) were acquired from Merck Life Science and used as received.

### 2.2 Synthesis and Characterization of MOF Materials and Device Fabrication

#### 2.2.1 Synthesis

All the MOFs were solvothermally synthesized following the methodology reported elsewhere with a slight modification.^47,48^ The details of the synthesis are described in the Supporting Information (SI, Section 1.1).

#### 2.2.2 Structural, Chemical, and Morphological Characterization

The MOF materials have been characterized by a combination of PXRD, Fourier-transformed infrared (FTIR) spectroscopy, total reflection X-ray fluorescence (TRXF), scanning electron microscopy (SEM), inductively coupled plasma optical emission spectroscopy (ICP-OES), and X-ray photoelectron spectroscopy (XPS). The related information is described in detail in the SI (Section 1.2).

#### 2.2.3 Steady-State Spectroscopic and Time-Resolved Photodynamics Characterizations

The steady-state spectroscopic and time-resolved photodynamical properties of the MOFs have been characterized by a combination of complimentary techniques, including an absorbance spectrophotometer, a fluorometer, time-correlated single photon counting, fs-transient absorption, and flash photolysis systems. The information is described in detail in the SI (Section 1.3).

#### 2.2.4 Photocatalytic and Photocurrent Characterization

The electrode fabrication along with the photoelectrochemical characterization and the photocatalytic tests is described in the SI (Section 1.4).

## 3 Results and Discussion

### 3.1 Structural, Chemical, Elemental, and Morphological Characterization of NDC-Ce(*x*) MOFs

**Scheme 1:**
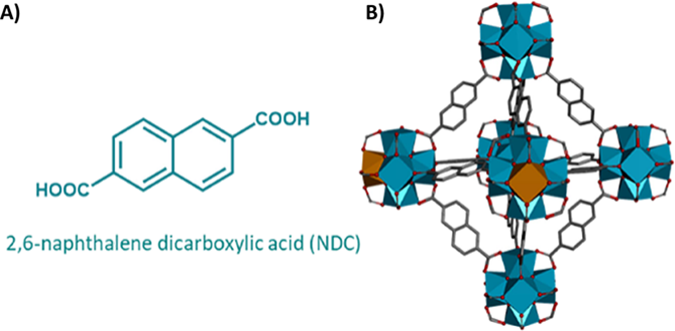
(A) Molecular Structure of the NDC Linker; (B) Schematic Illustration (Not in Scale) of the Crystalline Structure of Zr/Ce (NDC-Ce(*x*)) Mixed-Metal MOFs The blue polyhedral represents the Zr metal atoms in the clusters, while the orange polyhedral corresponds with Ce atoms acting as doping elements. This representation is for providing a visual idea of the replacement of Zr atoms by Ce in the clusters of the NDC-Ce MOFs with a content of Ce below 12%. Samples above 12% of Ce have an unknown crystalline phase (see the text).

Mixed-metal MOFs composed by Zr/Ce metal clusters in varying stoichiometric ratios and NDC organic linkers (labeled as NDC-Ce(*x*), where “*x*” denotes the percentage of the Ce metal in the MOF, *x* = 0, 2, 5, 9, 12, 25, 50, 75, and 100) have been designed and synthesized (Scheme 1). The structural, elemental composition, and morphological properties of the Zr/Ce containing NDC-Ce(*x*) MOF series were characterized by PXRD, FTIR, TXRF, ICP-OES, XPS, and SEM, respectively.

First, the percentage of Ce in the synthesized MOF structures was determined by TXRF. The obtained values were 1.9% of Ce for NDC-Ce (2), 5.2% for NDC-Ce (5), 9.6% for NDC-Ce (9), 11.7% for NDC-Ce (12), 25% for NDC-Ce (25), 50.2% for NDC-Ce (50), and 75% for NDC-Ce(75), respectively (Table S1 in the SI). These values show a good agreement between the amount of Ce used in the synthesis and the one found in the synthesized MOF structures. Additionally, we also performed ICP-OES experiments and compared the results with those calculated for theoretical Ce wt % of each material considering the chemical formula as C_72_H_36_O_32_Zr_6 – *x*_Ce_*x*_ (DUT-52). As depicted in Table S2, there is a good correlation between the calculated Ce wt% and the experimental values for the samples with a content of Ce below 12%. For the MOFs with amounts of Ce over 25%, there is a mismatch between the calculated and experimental values. This observation is explained considering the formation of a secondary phase (vide infra) with a distinct chemical formula to that of DUT-52-based MOFs used for the calculations. The results from TXRF and ICP-OES show a good agreement between the amount of Ce and Zr used during the synthesis and that found in the MOF material, reflecting that at low concentrations of Ce, the Ce atoms will most likely replace the Zr ones in the metal clusters. Hence, even though we cannot fully discard that some Ce atoms might coordinate with the metal nodes in the satellite, it is proposed that this will be a minimal fraction. If a high number of Ce atoms coordinate to the metal clusters, this will produce a mismatch in the ICP-OES results with the predicted formula, which is not the case. In any case, even if the Ce^IV^ location was not in full replacing nodal Zr^IV^ ions, the spectroscopic and photocatalytic properties of the mixed-metal MOFs would still be dominated by the photoinduced electron transfer from the NDC excited state to Ce^IV^. The XPS spectra of two MOFs, NDC-Ce(0) and NDC-Ce (9), were recorded (Figures S1 and S2 and Table S3 in the SI) and discussed in Section 3.2.

**Figure 1:**
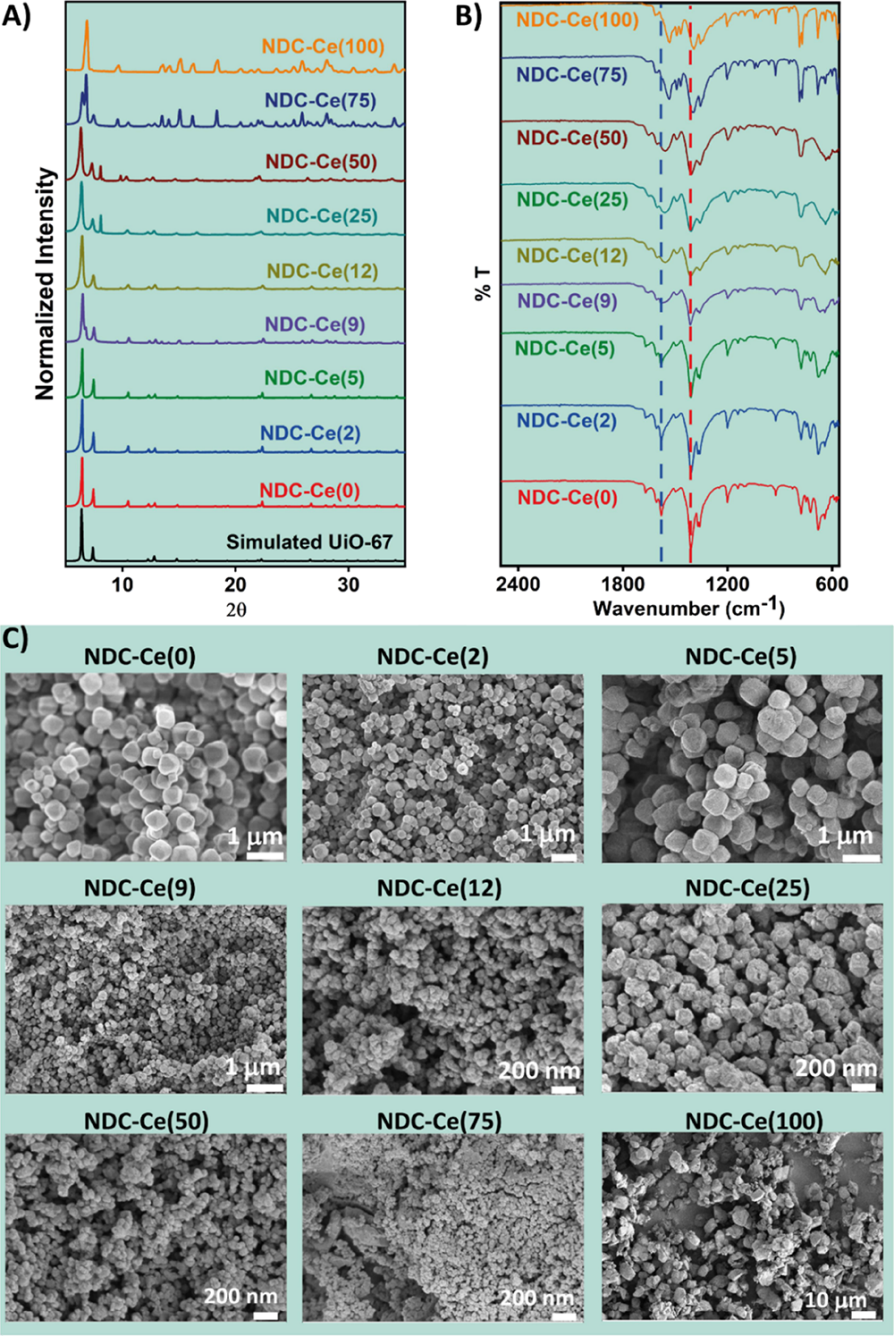
(A) Powder X-ray diffractograms, (B) FTIR spectra, and (C) SEM micrographs of NDC-Ce(*x*) MOFs (*x* = 0, 2, 5, 9, 12, 25, 50, 75, and 100).

The PXRD patterns show three distinct behaviors depending on the content of Ce (Figure 1A). For samples containing 0 to 9% of Ce, the PXRD patterns are coincident to those of the simulated PXRD pattern of DUT-52.^49^ As the percentage of Ce in these samples is low, the MOF materials maintain the same crystalline structure as the Zr-based DUT-52, and therefore, Ce can be considered to act as a dopant agent in the Zr clusters. On the other hand, when the amount of Ce rises to 12, 25, and 50%, although the PXRD patterns still display the characteristic peaks of DUT-52, new diffraction peaks (i.e., peaks at 8.1° and 9.9°) appear for these samples (Figure 1A). When the content of Ce in these MOFs is considerably large, we believe that under these conditions, the amount of Ce is enough to create new clusters that might interact with the NDC linker, yielding a secondary crystalline phase, different from the one observed for the DUT-52 isostructural MOFs. This is in agreement with previous reports, where Ce-ATA (ATA = amino terephthalic acid) and Zr/Ce-NDC-NH_2_ MOFs exhibit crystalline structure that differs from that of the Zr-based UiO-66.^50,51^ Indeed, these reported MOFs present diffraction peaks at ∼9.7° and 9.9°,^50,51^ similar to the one observed in our samples and attributed to a new undefined phase. Moreover, these observations are in good agreement with a previous report, where it was demonstrated via EXAFS experiments that when the amount of Ce in a Zr/Ce-UiO-66 MOF was lower than 17%, the Ce atoms replaced one Zr atom in the cluster, leading to the coexistence of bimetallic (i.e., CeZr_5_) cornerstones together with pure Zr_6_ clusters.^52^ On the other hand, when the content of Ce is above 17%, the Ce amount is significant enough to create Ce_6_ clusters.^52^ Hence, we propose that our samples behave similarly to the Zr/Ce-UiO-66 reported, in which a content of Ce below 12% creates CeZr_5_ clusters, while larger quantities of Ce induced the formation of Ce_6_ clusters, leading to the formation of another crystalline phase. Therefore, we suggest that the NDC-Ce (25) and NDC-Ce (50) MOFs are a combination of two crystalline structures resembling the DUT-52 (Zr-MOF) and NDC-Ce ones. Finally, the NDC-Ce(75) and NDC-Ce(100) MOFs show a completely different PXRD pattern, corroborating the formation of a Ce MOF with a crystalline phase different from that of Ce-DUT-52.^42^

These explanations of the PXRD patterns are further corroborated by the FTIR spectra of the MOFs recorded in the powder form (Figure 1B). A close inspection reveals two major changes in the FTIR spectra upon increasing the amount of Ce. On the one hand, a gradual change in the position is observed in the band located at 1580 cm^–1^ for NDC-Ce(0) that shifts up to 1536 cm^–1^ for the NDC-Ce(100) sample. On the other hand, the band located at 1405 cm^–1^ for NDC-Ce(0) also shifts up to 1392 cm^–1^ for NDC-Ce(100). Both shifts are more significant for the MOFs synthesized using percentages of Ce larger than 9%. These two bands correspond to the OCO asymmetric stretching (1580 cm^–1^) and symmetric stretching (1405 cm^–1^) vibrational modes of the carboxylate groups linked to the Zr clusters.^53^ Hence, the observed shifts in these particular vibrational modes indicate a change in the coordination environment of the linkers, as a consequence of their interaction with the Ce atoms, instead of the Zr ones. Similar behavior has been previously reported for a MOF-to-MOF transformation constructed with dicarboxylate linkers.^9^

The SEM images also correlate with the above conclusions (Figure 1C). The morphology and size of the MOF crystals having low content of Ce(*x* = 0–5) are very similar, showing an octahedral shape and a size around 0.3–0.5 μm, typical of UiO-type isostructural MOFs.^18,54−57^ The NDC-Ce(*x* = 9–12) crystals still preserve the octahedral morphology. However, the mean size of the crystals decreased to ∼100–200 nm. Even though the PXRD pattern of the NDC-Ce(12) sample shows the existence of a secondary NDC-Ce crystalline phase, the amount of the Ce-NDC MOF is not high enough to be perceived in the SEM images. Finally, the SEM images of the samples with higher percentages of Ce (NDC-Ce(*x* = 25–100)) show the loss of the octahedral morphology typical of UiO-type MOFs. These crystals are characterized by an irregular morphology that can be ascribed to the formation of the NDC-Ce phase having a different crystalline structure and morphology to that observed for the MOFs with low content of Ce.

### 3.2 Steady-State Absorption and Emission Spectra of NDC-Ce(*x*) MOFs

**Figure 2:**
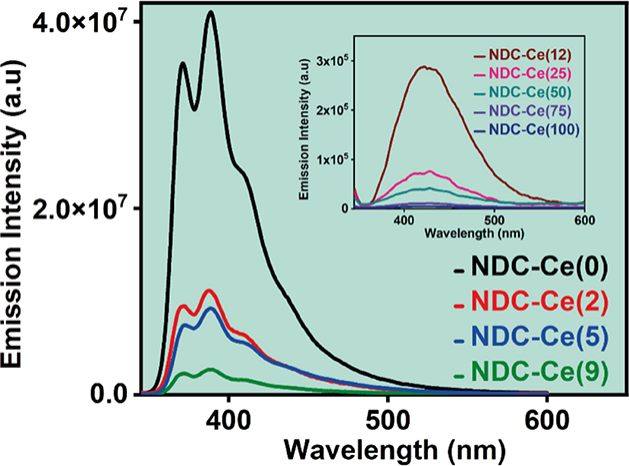
Emission spectra of the NDC-Ce(*x*) MOFs in DCM solvent. The inset is a zoomed-in (∼100 times) image of the emission spectra of NDC-Ce(12), NDC-Ce(25), NDC-Ce(50), NDC-Ce(75), and NDC-Ce(100). For the emission spectra, the excitation wavelength was 335 nm.

To begin with, we recorded the steady-state absorption and emission spectra of the MOFs in DCM. The absorption spectra of the MOFs consist of two structured absorption bands centered at 300 and 357 nm (Figure S3 in the SI). These vibrationally resolved bands are typical of the naphthalene linkers, as previously reported for other naphthalene-based Zr-MOFs.^43,58^ Similarly to these reported MOFs, the maximum in the absorption spectra compared to that of the linker in DCM is shifted by ∼10 nm (800 cm^–1^) toward longer wavelengths, suggesting a charge transfer interaction with the metal clusters.^43,44^ The increment of the absorption value at ∼300 nm might be a result of a stronger light dispersion by the MOF particles. The emission spectra of the MOFs upon photoexciting at 335 nm showed a clear dependence on the percentage of Ce with two distinct behaviors (Figure 2). The emission spectra of NDC-Ce(0), NDC-Ce(2), NDC-Ce(5), and NDC-Ce(9) are mirror images of their corresponding absorption spectra, with a vibrationally structured band having its maximum intensity at 390 nm, typical of naphthalene-based compounds. Similar to the absorption spectra, the maximum emission intensity of these MOFs is shifted by ∼15 nm (∼1025 cm^–1^) to longer wavelengths with respect to the pristine naphthalene-based linker, whose maximum is around 375 nm.^43^ The shift observed in the absorption and emission spectra of the naphthalene-based MOFs was ascribed to a charge transfer interaction with the metal clusters.^43,44^ Moreover, the emission spectra display a broad structureless shoulder with its maximum around 425 nm, ascribed to naphthalene excimer emission, in agreement with previous reports.^43,44^ Remarkably, the emission intensity decreased upon increasing of Ce in these MOFs. Doping of Zr-UiO-type MOFs with Ce atoms increases the LCCT interactions, owing to a better overlap between the π* orbitals of the linkers and those of the Ce atoms.^32^ Hence, we suggest that the increase in the Ce content in our MOFs enhances the LCCT process, inducing a quenching of their emission intensity. To further reinforce this assignment, we have performed XPS experiments of NDC-Ce(0) and NDC-Ce(9) MOF materials. As depicted in Figures S1 and S2 and Table S3, while the oxidation state of Zr is Zr^4+^ in all the samples, we can deduce that the NDC-Ce(9) material contains Ce atoms in both Ce^3+^ and Ce^4+^ oxidation states, evidenced by the existence of typical bands of Ce^4+^/Ce^3+^ (Figure S2E). The Ce^4+^/Ce^3+^ ratio in the NDC-Ce(9) material is almost 1/1. Therefore, the existence of Ce^4+^ atoms, which are highly reducible to Ce^3+^, favors the proposed LCCT mechanism in agreement with reported theoretical calculations.^32^

On the other hand, the emission spectra of NDC-Ce MOFs containing 12% of Ce onward are significantly different (Figure 2). They show a lack of vibrationally structured bands and consist of a broad structureless band with its maximum intensity at 425 nm, typical of naphthalene excimer emission. The change in the emission spectrum shape matches with the formation of a secondary crystalline phase in the samples containing more than 12% of Ce as described above. Since the naphthalene excimer formation strongly depends on the distance and orientation of the organic linkers, it is logical to attribute the change in the shape and position of the emission spectra to this secondary NDC-Ce phase, where the linkers should present a closer distance and a better orientation, thus favoring the excimer formation. Furthermore, the emission intensity of these MOFs gradually decreases with the Ce content, reflecting stronger charge transfer interactions with the Ce clusters. This is further corroborated with the photoluminescence quantum yields (PLQY) of these materials, which gradually decreases from 43% (NDC-Ce(0)) to 18% (NDC-Ce(2)), 16% (NDC-Ce(5)), 14% (NDC-Ce(9)), 3.4% (NDC-Ce(12)), 1.1% (NDC-Ce(25)), and 0.03% (NDC-Ce(50)). Note that the emission intensity of NDC-Ce(75) and NDC-Ce(100) was too weak to get an accurate value of their PLQY. A similar drop in PLQY was previously reported for Zr-based MOFs doped with Ce and Ce/Ti and ascribed to an enhancement of the LCCT process due to a more efficient overlap between the π*-orbitals of the BDC-NH_2_ linker with those of the metal cluster.^30^ Hence, the decrease in the emission intensity (i.e., PLQY values) with the increase in the Ce content is an indication of a more efficient LCCT photoreaction. However, for the samples having more than 12% of Ce, we cannot exclude that the secondary NDC-Ce phase might open new nonradiative deactivation pathways for the photoexcited NDC organic linkers, and consequently, the PLQY of the materials will also decrease.

Finally, the excitation spectra of all the studied MOFs in DCM suspensions are comparable to their absorption ones, indicating a common origin of the emitting species in the ground state (Figure S4, SI).

### 3.3 Picosecond Time-Resolved Emission Studies

**Figure 3:**
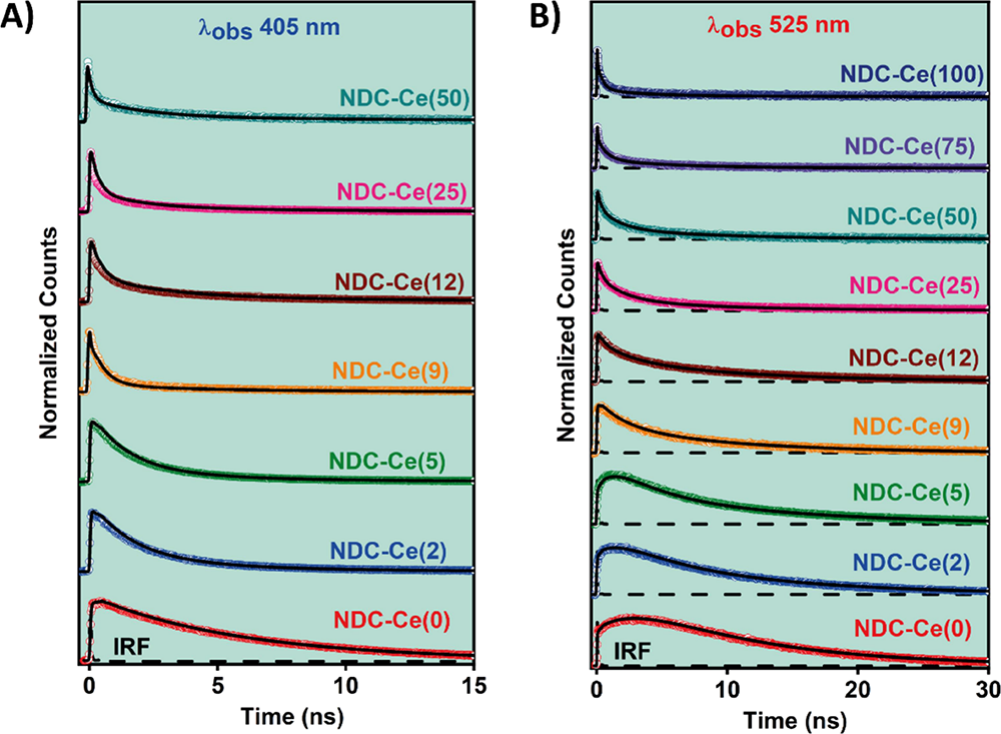
Magic angle emission decays of NDC-Ce(*x*) MOFs in a DCM suspension upon exciting at 371 nm and observing at (A) 405 nm and (B) 525 nm. Note that due to a very weak signal at 405 nm, the decays of NDC-Ce(75) and NDC-Ce(100) could not be recorded.

To understand the photodynamic processes occurring in the electronically first excited state (S_1_) of these MOFs, we recorded the ps–ns emission decays in DCM suspension using a 371 nm laser. The decays were recorded at different wavelengths spanning from 405 to 525 nm. Figure 3A,B displays the results at two selected wavelengths, 405 and 525 nm, while Table 2 depicts the time constants, relative amplitudes, and contributions obtained from a multiexponential analysis of these decays. Emission decays recorded in the range of 405–525 nm are presented in Figures S5–S13, while the corresponding fitting parameters from multiexponential fits are shown in Tables S4–S12.

To begin with, we briefly discuss the behavior of NDC-Ce(0) (equivalent to Zr-NDC (DUT-52)), as its photodynamics is well established (Figures 3 and S5).^43,44,46^ The fit gives three time constants of τ_1_ = 3.2 ns, τ_2_ = 7.0 ns, and τ_3_ = 14.5 ns (Tables 1 and S4). The intermediate (τ_2_) and longest (τ_3_) components are decaying in the whole spectral range; however, the τ_1_ component is decaying in the bluest emission region (405–435 nm, corresponding to the emission of naphthalene monomers), while it is rising (component with a negative amplitude) at wavelengths longer than 475 nm (emission region of naphthalene excimers). This type of decaying and rising components is typical of photoinduced processes happening at S_1_. Thus, we are recording the existence of two species sharing a common channel. Since the emission of the NDC excimers is shifted toward longer wavelengths, we attribute the longest component (τ_3_), whose contribution increases with the observation wavelength, to the emission lifetime of NDC excimers. On the contrary, the τ_2_ component exhibits its maximum contribution at the bluest wavelengths, and therefore, we assigned it to the emission of NDC monomers. Finally, the shortest τ_1_ component can be ascribed to the NDC excimer photoformation. This photodynamical behavior is similar to that reported for the pristine Zr-NDC MOF.^43,44,46^ However, the reported time components attributed to the excimer formation (τ_1_) and monomer lifetime (τ_2_) were much shorter than that for the NDC-Ce(0) MOF described herein. Given that the only difference between these two MOF materials is the use of water during the synthesis of NDC-Ce(0), we believe that water molecules might act as a modulator inducing defects in the final MOF structure, which can produce the observed changes in the excimer formation and monomer times. In fact, it has been demonstrated that the presence of water during the synthesis of the isostructural Zr-based UiO-66 MOF produces linker-missing defects in the structure. In this report, it was also proven that the emission properties of the MOFs depend on the number of defects in the material.^59^

**Table 1:** Values of Time Constants (τ_*i*_) and Normalized (to 100) Pre-exponential Factors (*a_i_*) and Contributions (*C_i_*) Obtained from the Fit of the Emission Decays of NDC-Ce(*x*) MOFs in DCM Solvent Recorded at 405 and 525 nm^a^

MOF	λ_obs_ (nm)	τ_1_ (ns)	*a*_1_	C_1_	τ_2_ (ns)	*a*_2_	C_2_	τ_3_ (ns)	*a*_3_	C_3_
**NDC-Ce(0)**	**405**	**3.2**	7	4	**7.0**	90	88	**14.5**	3	8
	**525**	**3.2**	–100	–100	**7.0**	58	42	**14.5**	42	58
**NDC-Ce(2)**	**405**	**2.0**	80	51	**5.1**	18	36	**13.0**	2	13
	**525**	**2.0**	–100	–100	**5.1**	60	28	**13.0**	40	72
**NDC-Ce(5)**	**405**	**1.5**	72	39	**3.2**	25	41	**13.0**	3	20
	**525**	**1.5**	–100	–100	**3.2**	55	20	**13.0**	45	80
**NDC-Ce(9)**	**405**	**0.5**	82	25	**2.5**	14	28	**11.4**	4	47
	**525**	**0.5**	5	1	**2.5**	45	17	**11.4**	50	82
**NDC-Ce(12)**	**405**	**0.4**	66	16	**3.0**	27	34	**12.3**	7	50
	**525**	**0.4**	27	2	**3.0**	35	18	**12.3**	38	80
**NDC-Ce(25)**	**405**	**0.3**	74	17	**1.8**	20	38	**8.4**	6	45
	**525**	**0.3**	43	4	**1.8**	40	30	**8.4**	17	66
**NDC-Ce(50)**	**405**	**0.3**	64	8	**1.6**	22	30	**9.5**	14	62
	**525**	**0.3**	47	4	**1.6**	36	24	**9.5**	17	72
**NDC-Ce(75)**	**405**	-	-	-	-	-	-	-	-	-
	**525**	**0.2**	64	5	**2.0**	30	28	**8.1**	6	67
**NDC-Ce(100)**	**405**	-	-	-	-	-	-	-	-	-
	**525**	**0.3**	80	18	**2.3**	15	38	**9.0**	5	44

a A negative sign indicates a rising component. The estimated uncertainty of the time constants, considering the errors from the experiments as well as those arising from the multiexponential fit of the signals, was between 10 and 15%.

The NDC-Ce(2) and NDC-Ce(5) MOFs exhibit emission decays similar to that described for NDC-Ce(0). Briefly, the decays were accurately fitted using a triexponential function, with a short component, which is decaying in the bluest spectral region and rising in the reddest one, and another two time components (τ_2_ and τ_3_) that are decaying in the whole emission spectral range (Figures 3, S6, and S7 and Tables 1, S5, and S6). Based on our previous discussion, these components are attributed to the excimer photoformation (τ_1_) and monomer (τ_2_) and excimer (τ_3_) lifetimes. Although the photodynamics of these MOFs is comparable to the one of NDC-Ce(0), it is worthy to note the decrease in the values of the time constants, especially of τ_1_ and τ_2_, which gradually decrease from 3.2 to 1.5 ns and from 7.0 to 3.2 ns, respectively. This change (Tables 1, S5, and S6) is an indication of the existence of new deactivation pathways that are competing against the excimer photoformation. In previous reports, it was demonstrated that the photoexcitation of the NDC linkers leads to an ultrafast LCCT phenomenon that may compete with the excimer formation. Consequently, the doping of the Zr clusters with Ce atoms enhances the orbital overlap with the naphthalene linkers, as explained above, and therefore, the LCCT becomes more favored. Since the LCCT is an ultrafast process,^30,44,45^ an increase in the efficiency of this phenomenon induces a decrease in the value of the risetime, as this value will be a combination of both processes. In addition, the decrease in the lifetime of the NDC monomers (from 7.0 to 3.2 ns) further corroborates the opening of additional nonradiative deactivation channels.

For the MOFs with Ce content larger than 9%, we observed slightly different photobehaviors (Figures 3 and S8–S13). Even though the emission decays of all these samples are accurately fit to a sum of three exponentials, all the components are decaying in the whole emission spectral range, and therefore, no rising component was recorded. The values of the time constants are τ_1_ = 0.2–0.5 ns, τ_2_ = 1.6–3.0 ns, and τ_3_ = 8.1–12.3 ns (Tables 1 and S7–S12). The τ_2_ and τ_3_ are ascribed to the emission lifetime of NDC monomers and excimers, respectively. The general trend is a decrease in these lifetimes upon increasing the content of Ce, reflecting the opening of a more efficient nonradiative deactivation pathway. This is in agreement with the quenching in the emission intensity observed for these samples (Figure 2). Moreover, the shorter component is now decaying in the whole spectral region, and therefore, this further indicates the competition between two different processes happening in the electronically first excited state of these MOFs. As stated above, LCCT and excimer formation are happening upon photoexcitation. Since the LCCT is an ultrafast process (<200 fs) and it becomes more efficient with the percentage of Ce in the samples, this will cause the disappearance of the rising component associated to the excimer formation. However, it is also possible that the NDC-Ce crystalline phase observed for the studied MOF samples with a content of Ce beyond 12% might affect the photodynamics of the materials, contributing to the observed quenching in the emission lifetimes. The following subsection will provide information about the ultrafast events in these MOFs.

### 3.4 fs-Transient Absorption Measurements

**Figure 4:**
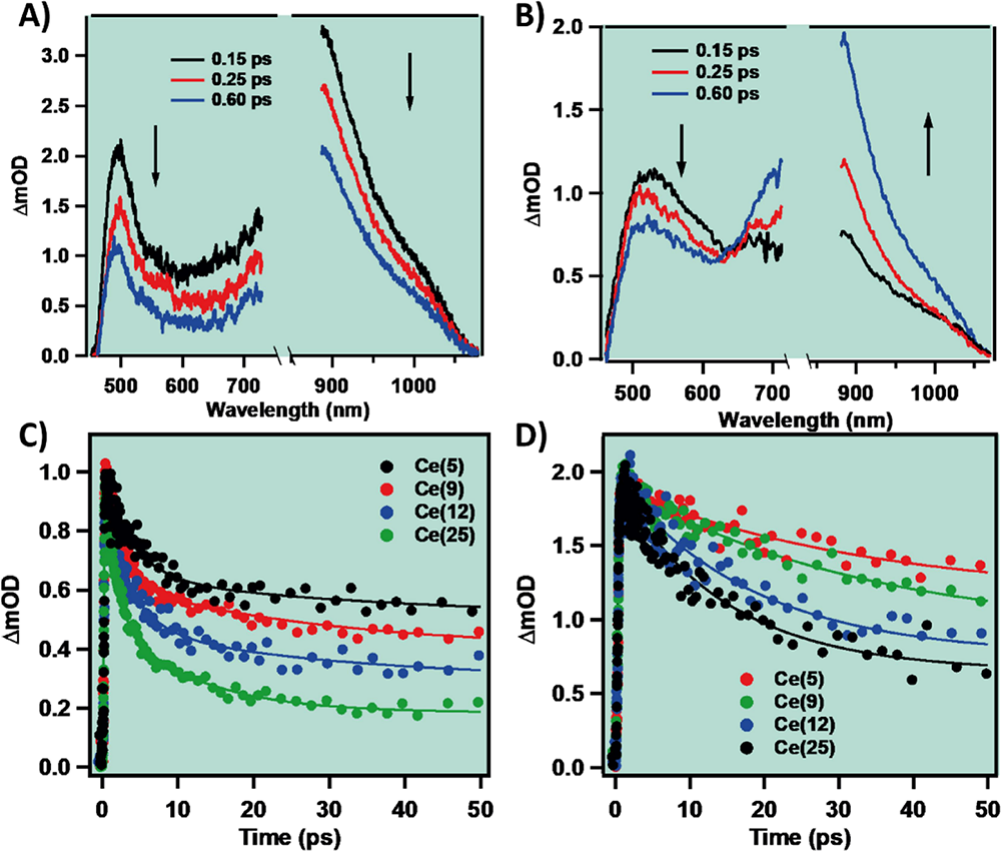
Time evolution of the transient absorption spectra of NDC-Ce(25) in (A) DCM and (B) DMF suspensions in terms of the change in the optical density (ΔmOD) upon excitation at 360 nm and recorded at the indicated delays. fs-TA decays of NDC-Ce(5), NDC-Ce(9), NDC-Ce(12), and NDC-Ce(25) in (C) DCM and (D) DMF suspensions gated at 900 nm following excitation at 360 nm. The instrument response function (IRF) was 120 fs. The solid lines in (C) and (D) represent the best multiexponential fit.

Thus, to gain insight into the effect of Ce incorporation on the nonradiative decay processes, we performed fs-visible-NIR-transient absorption (fs-TA) studies in DCM and DMF for NDC-Ce(5), NDC-Ce(9), NDC-Ce(12), and NDC-Ce(25). Figure 4A,B shows the fs-TA spectra (fs-TAS) at the early delay times for NDC-Ce(25). At the first glance, the studied four MOFs show similar TAS. In both solvents and for all the studied samples, the fs-TAS are composed of two positive bands centered at ∼500 and ∼900 nm. In DCM, both TA bands decrease in intensity with the delay time (Figure 4A), while in DMF, at early times (150–600 fs), the band at 500 nm decreases in intensity and the one at 900 nm concomitantly increases, suggesting a common ultrafast channel between the involved absorbing transient species (Figure 4B). At longer delay times the TAS decay in all the studied spectral range in both solvents, suggesting the same origin of the relaxation process. In agreement with previous reports on Zr-NDC MOFs, we assign the bands at 500 and 900 nm to the absorption of the excited NDC linkers and to the CSS produced following the LCCT reaction, respectively.^44,45^ To further characterize the ultrafast dynamics of the studied samples, we analyzed the transient decays collected at 900 nm (Figure 4C,D). The transients were fitted satisfactorily by a bi- (DCM) or triexponential (DMF) function (Table 2). In DCM, the fit of the transients gives two time components along with a constant contribution at longer delays. While the short component (∼3 ps) remains constant with the increase in the Ce content, the value of the second one decreases significantly from 38 ps for NDC-Ce(5) to 17 ps for NDC-Ce(25). Concomitantly, the contribution of the constant offset at longer delays also decreases with the increase in the Ce content. We observed similar trends in the behavior of the ps-time components for the transients collected in DMF giving time constants of τ_1_ ∼ 2 ps (for all MOFs) and τ_2_ = 34 ps for NDC-Ce(5) and 14 ps for NDC-Ce(25). In addition, in DMF, we found an ultrafast component that is decaying at 500 nm and rising at 900 nm with a value of ∼0.2 ps. We did not detect this component in the transients collected in DCM, which indicates that the related process is significantly faster in DCM (<100 fs). In agreement with previous report on Zr-based MOFs, we assign this time constant to an LCCT process giving rise to a CSS.^44^ The different values obtained in DCM and DMF were previously explained in terms of the more interactive nature of the DMF solvent molecules that can affect the electron orbital density around both the Zr node and the linker moieties and thus the electronic coupling involved in the CT formation.^44^ Notably, the value of this time constant remains unchanged in the decays of the studied samples, suggesting that the charge separation and LCCT processes are not affected by the Ce content. We assign the second component that has a value of 2–4 ps and is present in all the transients and in both solvents to the vibrational relaxation (VR) of the photoexcited linkers. Finally, the value of the longer ps-component shows higher sensitivity toward the amount of Ce in the MOF as its value systematically decreases (from 38 to 17 ps in DCM and from 34 to 14 ps in DMF for NDC-Ce(5) and NDC-Ce(25), respectively) with the increase of the Ce content. As reported previously for the pristine Zr-NDC MOF, this component arises from a fast nonradiative relaxation of the LCCT state to trap states.^44^ However, the sensitivity of this process to the presence of Ce and the decrease in the value of the related time constant suggest the opening of a new efficient nonradiative relaxation channel for the LCCT state due to Ce doping. The presence of more efficient nonradiative decay channels due to the Ce doping significantly affects the emission decay as observed in the steady-state and TCSPC measurements of the same systems (vide infra).

**Table 2:** Values of Time Constants (τ_*i*_) and Normalized (to 100) Pre-exponential Factors (*a_i_*) Obtained from the Fit of the fs-Transients of NDC-Ce(5), NDC-Ce(9), NDC-Ce(12), and NDC-Ce(25) in DCM and DMF Suspensions, at 360 nm Excitation and Gating at 900 nm; the Negative Sign Indicates the Rising Component

DCM						DMF						
MOF	τ_1_ (ps)	*a*_1_ (%)	τ_2_ (ps)	*a*_2_ (%)	a_3_ (%)	τ_0_ (ps)	*a*_0_	τ_1_ (ps)	a_1_ (%)	τ_2_ (ps)	*a*_2_ (%)	*a*_3_ (%)
**NDC-Ce(5)**	**2.80**	15	**38**	30	55	**0.20**	(−)100	**2.05**	10	**33**	40	50
**NDC-Ce(9)**	**2.72**	21	**35**	32	47	**0.23**	(−)100	**1.82**	12	**32**	48	40
**NDC-Ce(12)**	**2.93**	21	**22**	37	42	**0.21**	(−)100	**2.25**	15	**18**	48	37
**NDC-Ce(25)**	**3.05**	35	**17**	42	35	**0.18**	(−)100	**1.95**	18	**14**	53	29

### 3.5 μs-TA Flash Photolysis Observations

**Figure 5:**
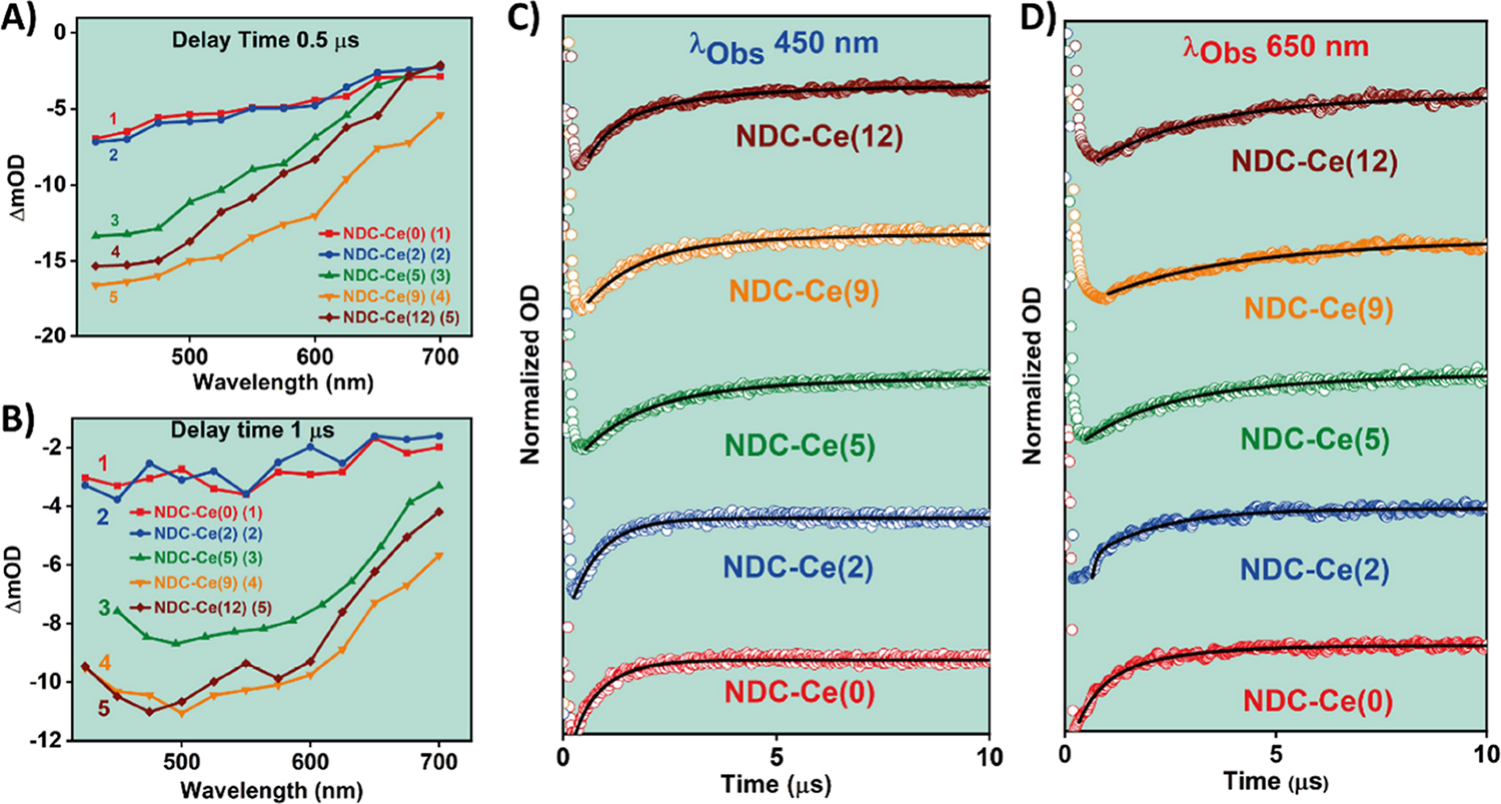
(A,B) μs-transient absorption spectra of NDC-Ce(0–12) MOFs in DCM suspensions upon exciting at 355 nm and collected at (A) 0.5 μs and (B) 1.0 μs delay times. (C,D) TA decays of NDC-Ce(0–12) MOFs in DCM suspensions recorded at (C) 450 nm and (D) 650 nm.

Once we have explored and discussed the ultrafast relaxation dynamics of the LCCT reactions in the selected MOF materials, their photodynamical properties in the microsecond temporal regime were explored to get information on the possible formation of long-lived CSSs. We focused on the samples having the following percentage of Ce: 0, 2, 5, 9, and 12. The samples with a content of Ce of 25% onward could not be accurately measured because of the strong light scatter interferences produced by the secondary NDC-Ce crystalline phase, which becomes more prominent (even predominant) for these MOFs. Figure 5A,B shows the TAS and decays of the NDC-Ce(0–12) MOFs in DCM suspensions, which were measured upon excitation at 355 nm, and represented at two delay times, corresponding to 0.5 and 1 μs. The TAS are characterized by a continuous negative band spanning from 700 to 400 nm, typical of systems having long-lived CSSs, as reported for several other MOFs.^30,34,44,45,60,61^ Interestingly, the TA intensity increases with the content of Ce in the MOFs up to NDC-Ce(9) and then it shows a slight decrease for the NDC-Ce(12). As the experimental conditions (concentration of the samples, excitation wavelength and power, temperature, etc.) have been kept the same for the five studied MOFs, the increase in the TA intensity reflects a more efficient formation of long-lived CSSs, as previously reported.^30,34^ The slight decrease in the TA intensity observed for NDC-Ce(12) could be explained by the appearance of the secondary NDC-Ce crystalline phase, in agreement with all of our previous results and discussion. Hence, we conclude that the generation of long-lived CSSs is more favored for NDC-Ce(9) than in the other Zr/Ce-based MOFs. This information is important for the potential use of these MOFs as photocatalysts, as it is well known that the formation of long-lived CSSs is one of the keystones for the photocatalytic reactions.

To get information on the lifetime of the CSSs, we recorded the TA decays for NDC-Ce(0–12) MOFs at 450 and 650 nm (Figures 5C,D). Figure S14A,B in the SI shows the decays at the full temporal scale at 450 and 650 nm, respectively. The values of the time constants (individual as well as averaged) and pre-exponential parameters obtained from the exponential analysis of these decays are depicted in Table 3. The transient decays of NDC-Ce(0) and NDC-Ce (2) are comparable, showing a monoexponential decay of ∼0.7 μs at the bluest region (450 nm) and a biexponential decay with times of ∼0.7 and ∼2 μs at the reddest one (650 nm). In line with previous reports, we assign the shorter component (∼0.7 μs) to the electron–hole (e^–^–h^+^) recombination from the shallow trap states, whereas the longer one (∼2 μs) is ascribed to e^–^–h^+^ recombination from deep trap states.^30,44,60,62^ With the increment in the percentage of Ce in the MOFs, the same biexponential nature of the decays was observed at both the blue and red spectral regions; however, the value of these time components is largely increased up to ∼3 and ∼20 μs, respectively (Table 3). Thus, in addition to the more efficient formation of CSSs for the MOFs with a higher amount of Ce (up to 9%), there is also an increase in the e^–^–h^+^ recombination time values, which is highly desired for photocatalytic applications as the CSSs are long-lived.

**Table 3:** Values of Time Constants (τ_*i*_) and Normalized (to 100) Pre-exponential Factors (*a_i_*) Obtained from the Fit of μs-Transient Decays of the NDC-Ce(0–12) MOFs in DCM Suspensions

MOF	λ_obs_ (nm)	τ_1_ (μs)	*a*_1_ (%)	τ _2_(μs)	*a*_2_ (%)	τ_av_ (μs)
NDC-Ce(0)	**450**	**0.7**	100	-	-	**0.7**
	**650**	**0.6**	62	**2.0**	**38**	**1.6**
NDC-Ce(2)	**450**	**0.8**	100	-	-	**0.8**
	**650**	**0.5**	65	**1.8**	35	**1.5**
NDC-Ce(5)	**450**	**1.5**	82	**6.8**	18	**4.1**
	**650**	**2.0**	70	**7.5**	30	**5.0**
NDC-Ce(9)	**450**	**1.4**	90	**11.0**	10	**6.0**
	**650**	**3.0**	75	**18.0**	25	**13.0**
NDC-Ce(12)	**450**	**1.0**	86	**3.0**	14	**2.0**
	**650**	**2.5**	80	**20.0**	20	**14.2**

**Scheme 2:**
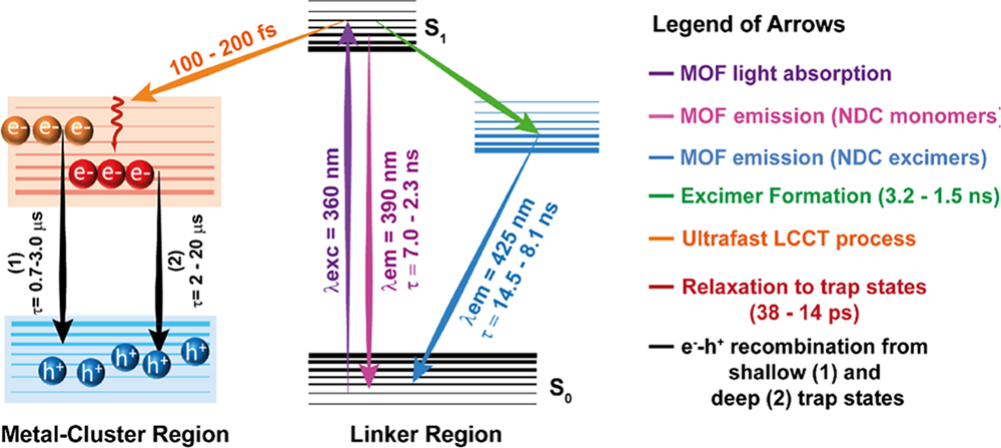
Schematic Illustration (Not in Scale) of the Different Photophysical Processes Occurring in the Electronically Excited States of the NDC-Ce(*x*) MOFs The data in purple, green, and sky-blue colors were obtained from the UV–vis steady-state and TCSPC experiments. The data in orange are from fs-TA, and the data in black are from the μs-TA experiments. Note that the increase in the content of Ce induces a general decrease in the time components obtained from TCSPC and fs-TA but an increase in the time of the e^–^–h^+^ recombination. Hence, the range in the values of the time constants represented in the scheme, corresponds with the increment of Ce in the MOFs.

Scheme 2 summarizes all the photophysical phenomena observed for these NDC-Ce(*x*) MOFs in a suspension of DCM. To conclude this section, we foresee that based on the photophysical results, the NDC-Ce(9) MOF will be the most promising material for photocatalytic applications, and to explore this possibility, we have performed photocurrent experiments of these MOFs.

### 3.6 Transient Photocurrent and Photoresponse

**Figure 6:**
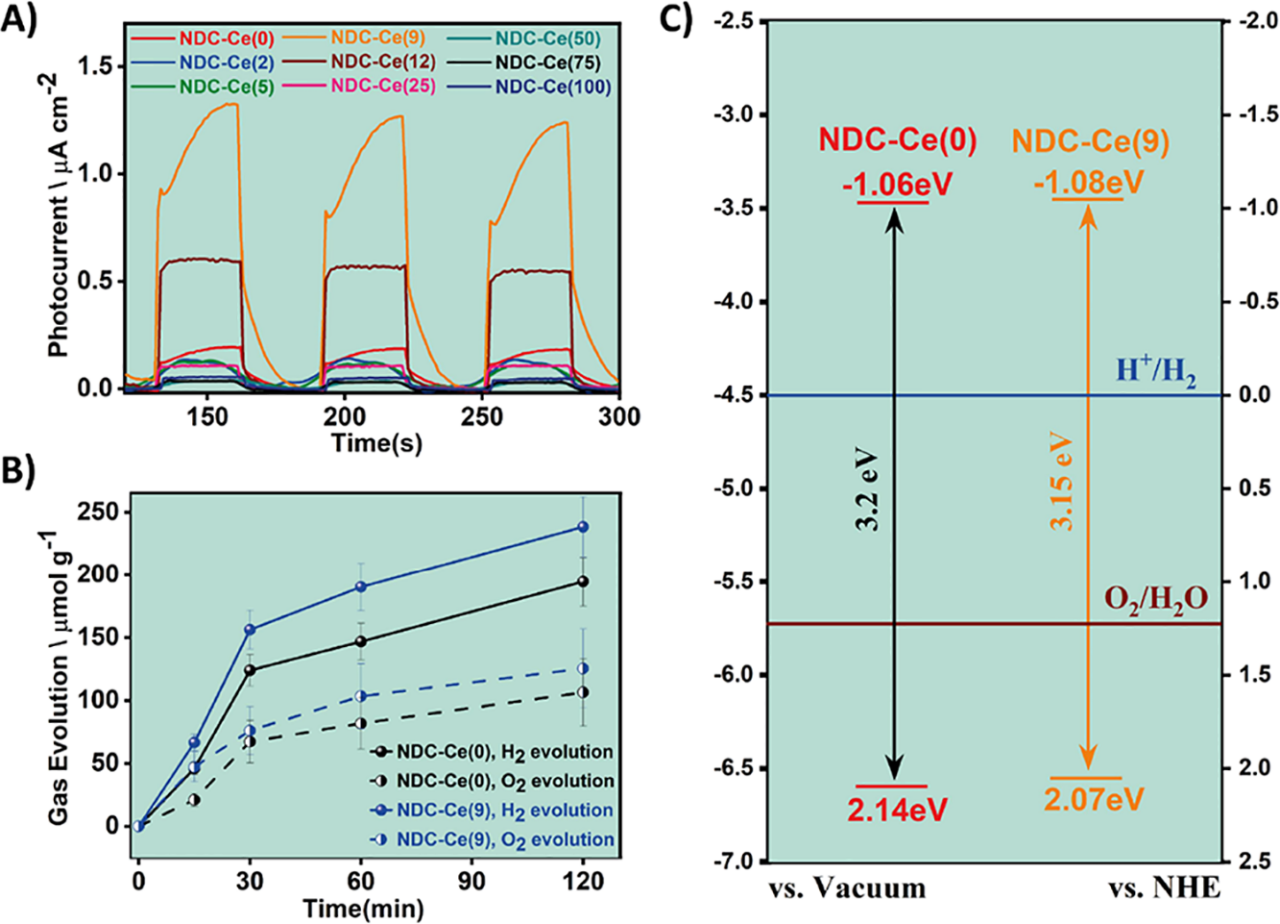
(A) Photocurrent vs. time measured from the NDC-Ce(*x*) photoelectrodes. A 300 W Xe lamp with a 400 nm cutoff filter was used as light source, as well as −0.3 V vs. Ag/AgCl applied bias. (B) Photocatalytic tests carried out employing NDC-Ce(0) (black) and NDC-Ce(9) (blue) with the time. The solid and dashed lines illustrate H_2_ and O_2_ evolution, respectively. (C) Values of the energy levels of NDC-Ce(0) and NDC-Ce(9) MOFs. The conduction band (CB) values were obtained via Mott–Schottky plots, while the valence band (VB) positions were determined by considering CB values and the optical band gap.

To begin with, Figure 6A shows the transient photocurrent results obtained upon irradiation at wavelengths larger than 400 nm with an applied bias potential of −0.3 V vs. Ag/AgCl. The studied MOFs were deposited on FTO substrates and used as working electrodes, while Pt wire and Ag/AgCl were used as counter and reference electrodes, respectively. The electrolyte was 0.5 M aqueous solution of Na_2_SO_4_. The dark current is very weak, and all electrodes containing NDC-Ce(*x*) MOFs exhibit photocurrent upon illumination, whose specific intensity depends on the Ce content. Figure S15 shows a plot of the maximum current density as a function of Ce percentage incorporated in the material, showing that there are two samples, NDC-Ce(9) and NDC-Ce(12), that clearly exhibit much higher photoresponse. This could be related with the quality of the MOF crystals and with the number of defects as well as the distribution of Ce^4+/3+^ in the particles. This influence, with the existence of an optimal Ce percentage, is not unexpected in photocatalysis since it has been frequently observed in other cases and attributed to the concomitant operation of opposite effects.^31^ On the one hand, an increase in Ce loading favors the photoresponse by promoting charge separation and Ce^3+^ acting as an electron trap, in agreement with our previous results. On the negative side, a high Ce percentage in the sample can introduce structural defects or induce the formation of a different crystalline phase (as described above) detrimental for the photocatalytic activity, since excessive Ce would possibly start acting as charge recombination centers. Regarding the shape of the transient photocurrent for NDC-Ce(9), the increase in the current density under constant illumination in the second timescale is probably due to charging effects, since the NDC-Ce(9) film can act as a battery/capacitor requiring intracrystalline ion diffusion to compensate photogenerated charge carrier migration. This ion migration can take place within seconds, both in the charging and discharging cycles.

The photoresponse was determined for NDC-Ce(0) and NDC-Ce(9) corresponding to the Zr-MOF without Ce and the sample exhibiting the highest transient photocurrent. Two types of measurements were performed: one using cutoff filters and the other one using a monochromator. In the first type of measurements, cutoff filters provide a visual idea of the relative contribution to the photoresponse of the spectral regions (Figure S17). Both samples, NDC-Ce(0) and NDC-Ce(9), exhibit a considerably higher photoresponse upon irradiation with the full UV/vis output of a 300 W Xe lamp (wavelength longer than 200 nm). By using a cutoff filter for deep UV light and allowing irradiation of the sample with UV A/B and visible light, a considerable decrease in the photoresponse is recorded for both MOF samples. This is due in part to a decrease in light power by filtering out the intense UV irradiation. Cutoff filters of a longer wavelength decrease gradually the intensity of the photocurrent, until it becomes negligible for wavelengths longer than 450 nm. Therefore, the visible light photoresponse of the samples arises only from the narrow wavelength range between 390 and 450 nm. Interestingly, the general behavior for NDC-Ce(0) and NDC-Ce(9) is the same, the photocurrent under any cutoff filter irradiation being higher for NDC-Ce(9) than for NDC-Ce(0). This reflects that the beneficial influence of the Ce loading does not arise from any specific spectral region. The above photocurrent response would agree with irradiation to the NDC ligand that upon excitation would transfer electrons to the metallic node as shown by the femtosecond and millisecond (flash photolysis) experiments. This electron transfer would be more efficient, allowing extraction of higher current densities, when Ce is present in the materials.

Similar tendency and photoresponse were also determined using monochromatic light. In the case of monochromatic light irradiation, external quantum efficiency values under specific electrode polarization could be measured. In particular, for the photoaction spectra, a more negative bias voltage of −0.5 V was employed in order to acquire higher signal-to-noise ratio signals. The photoaction spectra for NDC-Ce(0) and NDC-Ce(9) are presented in Figure S16A,B, respectively. Worth commenting is the much higher external quantum efficiency obtained for NDC-Ce(9), reaching a value of 0.65% at 360 nm.

**Scheme 3:**
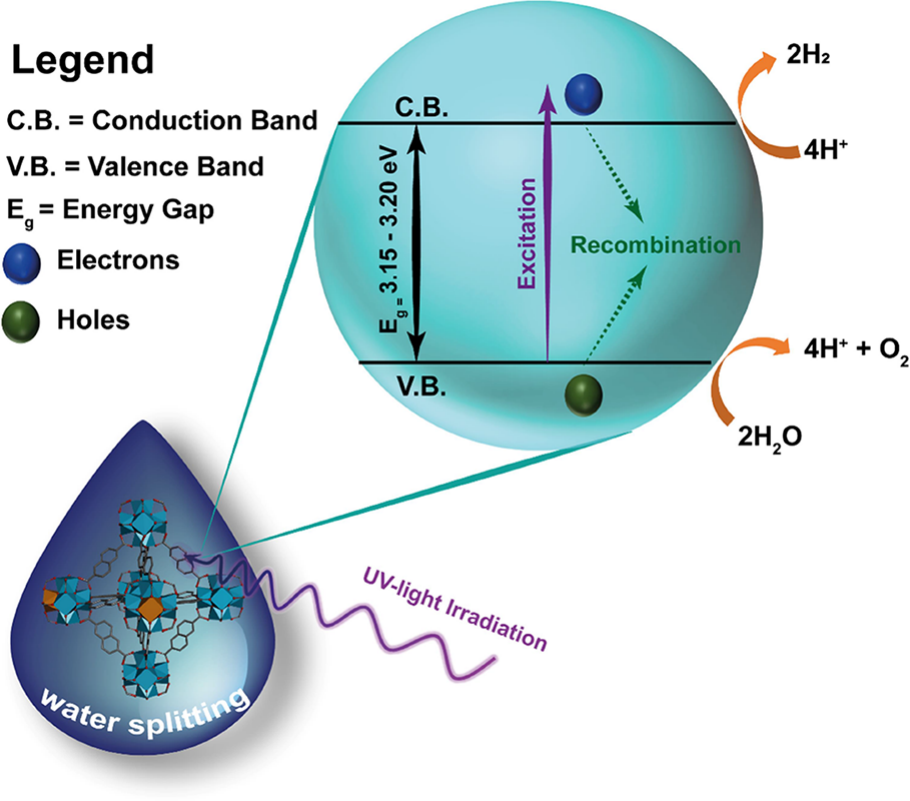
Representation of the Photocatalytic Water Splitting Using the NDC-Ce(0) and NDC-Ce(9) MOFs The representation of the MOF is for providing a visual idea of the replacement of Zr atoms by Ce in the clusters of the NDC-Ce MOFs with a content of Ce below 12%.

The photocatalytic activities of NDC-Ce(0) and NDC-Ce(9) were assessed in the overall water splitting reaction using distilled water in the absence of sacrificial electron donors. The time-dependent evolution of both H_2_ and O_2_ is illustrated in Figure 6B and the initial H_2_ evolution for NDC-Ce(0) and NDC-Ce(9) is shown in Figure S18. After 2 h of irradiation with UV–visible light, NDC-Ce(0) and NDC-Ce(9) evolve 194 and 238 μmol·g^–1^ H_2_, respectively, with O_2_ evolution close to the stoichiometric amount. The reaction kinetics show two distinct linear domains, the fastest one corresponding to a H_2_ evolution rate of 247 μmol·g^–1^·h^–1^ for NDC-Ce(0) and 312 μmol·g^–1^·h^–1^ for NDC-Ce(9), respectively, equivalent to an enhancement in photocatalytic activity of around 20% upon Ce incorporation. Scheme 3 outlines the general scheme of the photocatalytic water splitting reaction.

Postreaction solutions were thereafter analyzed by ICP-OES for metal leaching. No traces of Zr could be observed for both samples, yet for the NDC-Ce(9) sample, 0.9 wt % Ce was detected in the aqueous solution after irradiation.

Nyquist plots of NDC-Ce(0) and NDC-Ce(9) were also measured under irradiation using the same electrolyte (Figure S19). The smaller radius of NDC-Ce(9) clearly shows that charge migration and mobility in NDC-Ce(9) under illumination is much higher in this sample than in NDC-Ce(0). This information indicates that in addition to the elementary step of photoinduced electron–hole separation, ion migration is also favored when Ce is present in the material, in line with the observed battery/capacitor behavior observed for NDC-Ce(9) in the transient photocurrent measurements. Mott–Schottky plots considering the MOFs as n-type semiconductors were also measured to determine the CB potential of the reference NDC-Ce(0) and of NDC-Ce(9) as presented in Figure S20. A visual representation of the band positions is shown in Figure 6C. Compared to the NDC-Ce(0) sample, NDC-Ce(9) shows a negligible shift in the positions of both the CB and VB. Hence, the inclusion of Ce in the MOF structure at this small loading level does not directly influence the reductive/oxidative capacity of the semiconductive material. As previously discussed, Ce incorporation at higher percentages increases the e^–^–h^+^ recombination due to the generation of additional trap states; hence, there is an optimal Ce percentage to achieve the highest photocatalytic activity.

## 4 Conclusions

Herein, we have unveiled which is the optimal content of Ce in naphthalene-based Zr/Ce MMOFs for photocatalytic applications by a complimentary combination of spectroscopic, photodynamic, and photoactivity measurements. To this end, we have synthesized a series of MOFs in which the ratio of Ce was augmented from 0, 2, 5, 9, 12, 25, 50, 75, and 100%. The structural and chemical characterization indicates that for the MOFs with 12% Ce onward, in addition to the UiO crystalline structure, a secondary crystalline phase, attributed to a NDC-Ce MOF, was cosynthesized. The emission spectra of the MOFs strongly depend on the percentage of Ce, varying from a vibrationally resolved band observed for the MOFs containing 0–9% to a broad red-shifted structureless band obtained for the MOFs with 12–100% of Ce. This difference in the emission spectra is attributed to the formation of the NDC-Ce crystalline phase. The photodynamic properties of these MOFs reflect the existence of two competing photoprocesses that are affected by the content of Ce. These photoprocesses are the excimer formation between the naphthalene linkers and an ultrafast LCCT happening in a fs-time scale upon the photoexcitation of the organic linkers. The increase in the Ce content favors the LCCT process over the excimer photoformation owing to the low-lying empty 4f orbitals of Ce^4+^ that improve the overlap with the π*-orbitals of the organic linkers. This induced a quenching of the photoluminescence quantum yield as well as a decrease in the fluorescence lifetimes. Furthermore, the LCCT process yields the formation of long-lived CSSs, which has been demonstrated to be more effective for NDC-Ce(9), thus pointing out that this MOF would behave as the most efficient photocatalyst in this family. To corroborate this assumption, we performed transient photocurrent experiments and proved that the MOF with 9% of Ce exhibits the highest photocurrent of this series of MOFs. As a proof of concept, photocatalytic water splitting reaction was performed using NDC-Ce(0) and NDC-Ce(9), where the results showed that the overall efficiency of the process is enhanced by 20% when the MOF is doped with 9% of Ce compared with the pristine Zr-NDC MOF. Using the results of (i) a combination of structural and morphological characterizations, (ii) spectroscopic and fs–ms time-resolved experiments, and (iii) correlating them with the photocurrent activity and water splitting applications, we provide clear findings to better design mixed Zr/Ce metal MOFs for sustainable photocatalytic applications.

## Supporting Information Available

Detailed information about the synthesis and methodology; table for Zr and Ce composition as determined from TXRF measurements; table for Ce composition as determined from ICP-OES measurements; XPS data for NDC-Ce(0) and NDC-Ce(9); table for orbital energies obtained from XPS measurements for NDC-Ce(0) and NDC-Ce(9); the absorption and excitation spectra of NDC-Ce(*x*) samples in DCM suspension; the emission decay traces of NDC-Ce(*x*) samples in DCM suspension; tables of the parameters obtained from the fitting of the decay traces of NDC-Ce(*x*) MOFs in DCM suspension; μs-TA decays of NDC-Ce(0–12) MOFs in DCM at two wavelengths in the full temporal scale; the photoaction plot of NDC-Ce MOFs; photocurrent measurements of NDC-Ce(0) and NDC-Ce(9) electrodes using cutoff irradiation; initial H_2_ evolution rates of the NDC-Ce(0) and NDC-Ce(9) photocatalysts; Nyquist plots for NDC-Ce(0) and NDC-Ce(9); Mott–Schottky plots for NDC-Ce(0) and NDC-Ce(9); and references (PDF)
