# Supplementary material for: Unraveling the Optimal Cerium Content for Boosting the Photoresponse Activity of Mixed-Metal Zr/Ce-Based Metal–Organic Frameworks through a Photodynamic and Photocurrent Correlation: Implications on Water Splitting Efficiency
Source: ACS Appl Mater Interfaces. 2023 Jul 21;15(30):36434–46. doi: 10.1021/acsami.3c08062 (PMC10401508; doi:10.1021/acsami.3c08062)
Supplement: Supplementary file 1 — am3c08062_si_001.pdf [file am3c08062_si_001.pdf]

Supporting Information

for

Unravelling the Optimal Cerium Content for Boosting the Photoresponse Activity of Mixed Metal Zr/Ce Based MOFs through a Photodynamic and Photocurrent Correlation: Implications on Water Splitting Efficiency

Arghyadeep Bhattacharyya,[1‡] Mario Gutiérrez,[1‡] Boiko Cohen,[1] Horatiu Szalad,[2] Josep Albero,[2] Hermenegildo Garcia,[2] Abderrazzak Douhal[1*]*

[1]Departamento de Química Física, Facultad de Ciencias Ambientales y Bioquímica, and INAMOL, Universidad de Castilla-La Mancha, Avenida Carlos III, S.N., 45071 Toledo, Spain. *Email: Abderrazzak.Douhal@uclm.es*

[2]Instituto de Tecnología Química, Consejo Superior de Investigaciones Científicas-Universitat Politecnica de Valencia, Universitat Politecnica de Valencia, Av. De los Naranjos s/n, 46022 Valencia, Spain. *Email: hgarcia@qim.upv.es*

[‡]*Equal Contribution*

[*]*Corresponding authors*

Contents

1. Methodology

1.1. Synthesis of MOF Materials

1.2. Structural, Chemical and Morphological Characterization of MOF Materials

1.3. Steady-State Spectroscopic & Time-Resolved Photodynamics Characterization

1.4. Photocurrent Characterization

2. Table for Zr and Ce composition as determined from TXRF measurements.

3. Table for Ce composition as determined from ICP-OES measurements.

4. XPS data for NDC-Ce(0) and NDC-Ce(9).

5. Table for orbital energies obtained from XPS measurements for NDC-Ce(0) and NDC-Ce(9).

6. Absorption Spectra of NDC-Ce(x)s in DCM suspensions.

7. Excitation spectra of NDC-Ce(x)s in DCM suspension.

8. Emission decay traces of NDC-Ce(x)s in DCM suspension.

9. Tables of the parameters obtained from the fitting of the decay traces of NDC-Ce(x) MOFs in DCM suspension.

10. μs-TA decays of NDC-Ce(0-12) MOFs in DCM at two wavelengths in full temporal scale.

11. The photo action plot of NDC-Ce MOFs.

12. Photocurrent measurements of NDC-Ce (0) electrodes and NDC-Ce(9) using cut-off irradiation.

13. Initial H_2 evolution rates of the NDC-Ce (0) and NDC-Ce (9).

14. Nyquist plots for NDC-Ce (0) and NDC-Ce (9).

15. Mott-Schottky plots for NDC-Ce (0) and NDC-Ce (9).

16. References

1. Methodology

1.1. Synthesis of MOF Materials

Synthesis: All the MOFs were solvothermally synthesized following the methodology reported elsewhere with a slight modification.[1,2] Briefly, 0.13 g (0.6 mmol) of napthalene-2,6-dicarboxylic acid (NDC), 15 mL of DMF and 1 mL of glacial acetic acid were mixed in a 30 mL glass vial. The mixture was heated to 100 ºC until the complete dissolution of the linker. Afterwards, 1.0 mL of an aqueous solution of the metal salt(s) in equimolar ratio with respect to the linker (i.e, 0.6 mmol) and with different ratios of $ZrCl_4$ and $(NH_4)_2[Ce(NO_3)_6]$ (100/0, 98/2, 95/5, 88/12, 75/25, 50/50, 25/75, 0/100 of Zr/Ce) were added to the hot solution of the linker. The vials were sealed, put into a preheated oven, and maintained at 120ºC for 24 hours. Then, the colloidal suspensions were cooled to room temperature, centrifuged, and thoroughly washed with DMF until the supernatant does not show any emission of the linker, suggesting a complete removal of unreacted linkers. The powdery MOF samples were collected by centrifugation and dried at 120 ºC overnight. The MOFs were labelled according to the amount of Ce used in the synthesis, i.e., NDC-Ce(x) where x denotes the percentage of the Ce metal in the MOF cluster (x = 0, 2, 5, 9, 12, 25, 50, 75 and 100). The mass of MOF obtained for all the reactions was between 0.23 - 0.35 g.

1.2. Structural, Chemical and Morphological Characterization of MOF Materials

The powder X-ray diffraction (PXRD) patterns of MOF powders were obtained using a PANalytical diffractometer (X'Pert Pro model) and a X Bruker D8 Advance. The conditions used were 45 kV, 40 mA, CuKα radiation, and a system of slits (soller–mask–divergence–antiscatter) of 0.04 rad–10 mm–1/8°–1/4° with a X'celerator detector.

The Fourier-transformed infrared (FTIR) spectra of the MOFs were recorded using a Perkin-Elmer Spectrum 100 equipped with Mid-IR deuterated triglycine sulphate, mercury cadmium telluride detectors and with universal attenuated total reflection.

Elemental analyses of MOFs were performed by a total reflection X-ray fluorescence (TXRF) Bruker S2 PICOFOX instrument, using Mo K radiation with a count rate of 3159 cps, current density of 600 μA and employing a voltage difference of 50 kV.

The scanning electron microscopy (SEM) images of the MOFs were collected by using a field emission scanning electron microscope (Zeiss GeminiSEM 500, Oberkochen,

Germany) operating in high vacuum mode. The MOF samples were mounted onto standard aluminum SEM stubs using conductive carbon adhesive tabs and coated with gold.

XPS data were obtained through a SPECS spectrometer with a Phoibos 150 MCD-9 detector. The Al and Mg nonmonochromatic X-ray sources were operated at 200W. Before sample analysis and data acquisition, the XPS antechamber was evacuated under vacuum at 10^{-9} mba. The work function of the device was calibrated with Ag, Au, and Cu standards resulting in a value of 4.2440 eV. The measured intensity ratios of the analyzed components were obtained from the area of the corresponding peaks after nonlinear Shirley-type background subtraction and corrected by the transition function of the spectrometer.

The Ce contents of all NDC-Ce samples were determined through inductively coupled plasma-optical emission spectrophotometry by employ of a Varian 715-ES (CA, USA) device. All samples were digested in an acidic mixture of HCl and HNO_3 (3:1 v/v). Before quantification, a calibration with standards of known concentrations was carried out.

1.3. Steady-State Spectroscopic & Time-Resolved Photodynamics Characterization

The steady-state UV-visible absorption and emission spectroscopic measurements were carried out using a JASCO V-670 and a FluoroMax-4 (Jobin-Yvone) spectrophotometers, respectively. The FluoroMax-4 spectrophotometer is equipped with a Quanta integrating-sphere setup from Horiba allowing for measuring the photoluminescence quantum yield (PLQY) of the MOF samples.

The picosecond (ps) time-resolved emission experiments were carried out by employing a time-correlated single-photon counting (TSCPC) system. The samples were excited by a 40 ps-pulsed (<1 mW, 40 MHz repetition rate) diode-laser (PicoQuant) centred at 371 nm. The instrumental response function (IRF) is around ~70 ps. The fluorescence signal was gated at the magic angle (54.71°) and monitored at a 90° angle to the excitation beam at discrete emission wavelengths. The decays were deconvoluted and fitted to a multiexponential function using the Fluofit package (PicoQuant) allowing single and global fits. The quality of the fits, as well as the number of exponentials, were carefully evaluated based on the reduced χ^2 values (which were always below 1.3) and the distributions of the residuals.

The nanosecond (ns) flash photolysis setup has been described previously.[3] Briefly, it consists of an LKS.60 laser flash photolysis spectrometer (Applied Photophysics) and a Vibrant (HE) 355 II laser (Opotek). To excite the sample, we used the third harmonic (355 nm) output. The sample was pumped with a fluence of 2.1 mJ/cm^2. As a probe source, we used the output of a 150 W Xenon arc lamp. The light transmitted through the sample was then dispersed by a monochromator and detected by a visible photomultiplier (Applied Photophysics R928), coupled to a digital oscilloscope (Agilent Infiniium DS08064A, 600 MHz, 4 GSa s1). The measured IRF of the system was ~8 ns. All the experiments were performed at 293 K and atmospheric conditions. Moreover, the photostability of the MOFs was tested by measuring the absorption and emission spectra before and after the time-resolved experiments, where the samples were continuously pumped by the lasers described above. We observed no significant changes in any of the spectra, reflecting the high photostability of these MOFs under the used experimental conditions.

For the steady-state, TCSPC and flash photolysis experiments, we prepared 3 mL suspensions of each MOF in dichloromethane (DCM) adjusting the optical density to a value of 0.3 at 357 nm (1 cm pathway). This corresponds to ~1.0-1.2 mg of MOF in 3 mL of DCM.

The fs transient UV−vis−NIR absorption setup has been described elsewhere.[4] Briefly, it consists of a Ti: Sapphire oscillator (TISSA 50, CDP Systems) pumped by a 5 W diode laser (Verdi 5, Coherent). The seed pulse (30 fs, 450 mW at 86 MHz) centred at 800 nm is directed to chirped pulse amplification system (Legend-USP, Coherent). The amplified fundamental beam (50 fs, ~3 W at 1 kHz) is then split by a beam splitter and the main portion (2.7 W) is directed through an optical parametric amplifier for wavelength conversion (TOPAS, Light Conversion). A small portion of the rest of the fundamental beam (~200 μW) is used for white light continuum generation in a 3-mm thick sapphire crystal. The used pump intensity was ∼500 μW (spot size at the sample was 280 μm) and the excitation wavelength was 360 nm. The instrumental response function (IRF) was 120 fs. Transient absorption measurements were performed in the spectral ranges of 430−620 (UV−vis region) and 870 – 1050 nm (NIR). To avoid sample degradation, the samples were placed in a 0.5 mm rotating cell. The data were analysed using a multiexponential global fit. The quality of the global fit was checked by examining the residual distributions. The estimated error is 10 – 15%.

1.4. Photocurrent Characterization

Electrode preparation: All NDC-Ce samples were dispersed in a mixture containing ethanol and a Nafion binder and subsequently drop casted on FTO glass electrodes. The solvent was allowed to evaporate before electrode testing.

Photoelectrochemical characterization: All measurements employed Ag/AgCl a platinum wire as reference and counter electrodes, respectively. A 0.5M aqueous solution of Na_2SO_4 was used as electrolyte. All photoelectrochemical data were acquired via a VersaStat3 potentiostat. The irradiation source was a 300 W Xe Lamp (0.1 W/cm^2 light intensity) attached with different cut-off filters. The electrochemical cell was irradiated with an optical fibre fixed at 2 cm from the cell.

Photo action data were recorded by irradiating the cell with a 300 W Xe lamp source light passing through a triple grating monochromator from Optics Focus. The light intensities were measured with a photodiode system from Hamamatsu.

External quantum efficiency values (EQE) were calculated employing the following formulae:

$$SR(\lambda) = \frac{I(\lambda)}{P(\lambda)} \text{ in } (A/W); \qquad EQE(\lambda)\% = \frac{1240 \cdot SR(\lambda)}{\lambda};$$

where SR(λ) is the spectral response, I(λ) is the photocurrent recorded by the system upon light irradiation, P(λ) is the power of incident irradiation at each wavelength and λ is the wavelength of the incident photons.

Nyquist plots were determined in the frequency range between 10^6 Hz to 0.01 Hz.

Photocatalytic Tests: A cylindrical quartz reactor attached with two valves (inlet and outlet and a manometer) was loaded with a 1 mg/mL aqueous dispersion of NDC-Ce MOFs and flushed with pure Ar gas for 15 min. The reactor was thereafter degassed for 15 min via bath sonication and flushed with argon for additional 30 min. The photocatalytic reactions were carried out under irradiation from a 300 W Xe lamp (0.1 W/cm^2 light intensity) without applying any external heat source.

The evolving gases (H_2 and O_2) were monitored with a Agilent490 MicroGC equipped with a MolSieve 5Å column (Ar carrier gas) and a TC detector. Gas quantification was carried out by a calibration plot using commercial H_2 mixtures of known composition.

2. Table for Zr and Ce Composition as determined from TXRF measurements.

Table S1. Percentages of Zr and Ce elements found in the NDC-Ce(x) MOFs measured by TXRF spectroscopy.

MOF	% of Zr	% of Ce
**NDC-Ce(2)**	98.1	1.9
**NDC-Ce(5)**	94.8	5.2
**NDC-Ce(9)**	90.4	9.6
**NDC-Ce(12)**	88.3	11.7
**NDC-Ce(25)**	75.0	25.0
**NDC-Ce(50)**	49.8	50.2
**NDC-Ce(75)**	25.0	75.0

3. Table for Ce composition as determined from ICP-OES measurements.

Table S2. Ce wt.% found for the MOF samples determined from ICP-OES measurements and the values calculated using the chemical formula $C_{72}H_{36}O_{32}Zr_{6-x}Ce_{x}$.

Sample	Ce wt.% Found	Ce wt.% calculated from $C_{72}H_{36}O_{32}Zr_{6-x}Ce_{x}$
NDC-Ce(0)	0.08	0.00
NDC-Ce(2)	0.93	1.32
NDC-Ce(5)	0.98	1.65
NDC-Ce(9)	3.29	3.80
NDC-Ce(12)	5.21	5.05
NDC-Ce(25)	9.41	10.3
NDC-Ce(50)	12.17	19.9
NDC-Ce(75)	22.07	29.9
NDC-Ce(100)	30.19	37.3

4. XPS data for NDC-Ce(0) and NDC-Ce(9).

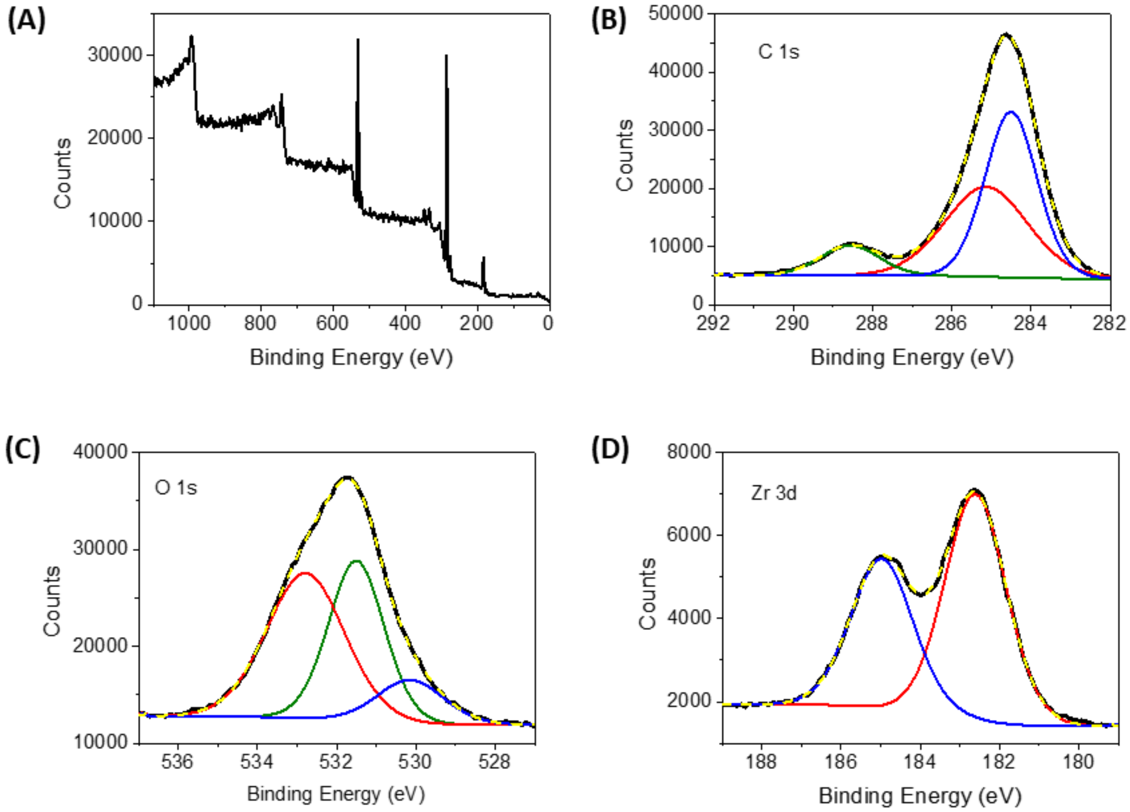

Figure S1: (A) Survey spectrum of NDC-Ce(0). High Resolution XPS scans of **(B)** C 1s, **(C)** O 1s and **(D)** Zr 3d for NDC-Ce(0).

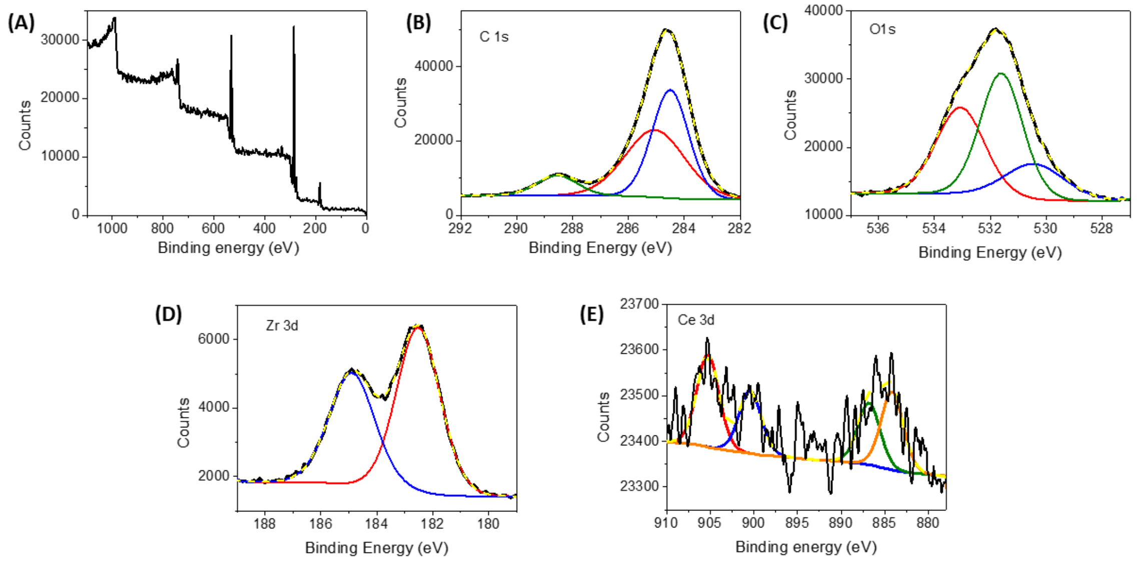

Figure S2: (A) Survey spectrum of NDC-Ce(9). High Resolution XPS scans of **(B)** C 1s, **(C)** O 1s, **(D)** Zr 3d and **(E)** Ce 3d for NDC-Ce(9).

5. Table for orbital energies obtained from XPS measurements for NDC-Ce(0) and NDC-Ce(9).

Table S3. Orbital energies as obtained from XPS measurements for NDC-Ce(0) and NDC-Ce(9).

MOF	Bond/Orbital type	Energy (ev)
**NDC-Ce(0)**	C=C	285.5
	C-C	285.1
	O-C=O	288.1
	M-O	530.15
	O-C	531.50
	O=C	532.78
	Zr $3d_{5/2}$	182.60
	Zr $3d_{3/2}$	184.96
**NDC-Ce(9)**	C=C	284.50
	C-C	285.05
	O-C=O	288.55
	M-O	530.44
	O-C	531.60
	O=C	533.06
	Zr $3d_{5/2}$	182.51
	Zr $3d_{3/2}$	184.89
	$Ce^{3+}3d_{3/2}$	905.26
	$Ce^{4+}3d_{5/2}$	900.42
	$Ce^{4+}3d_{3/2}$	886.82
	$Ce^{3+}3d_{5/2}$	884.16

6. Absorption spectra of NDC-Ce(x)s in DCM suspension.

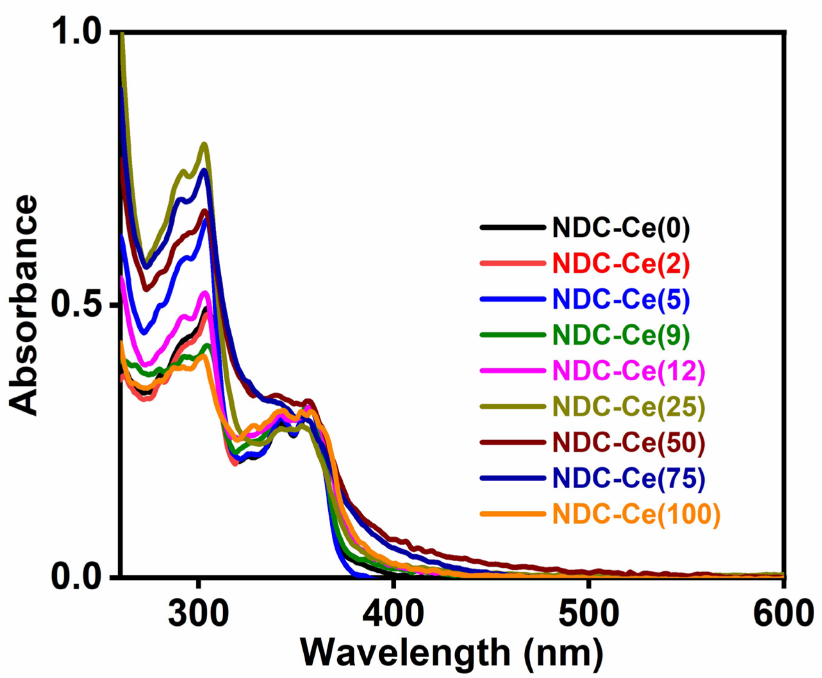

Figure S3: Absorption spectra of NDC-Ce(x)s in DCM suspension.

7. Excitation spectra of NDC-Ce(x)s in DCM suspension.

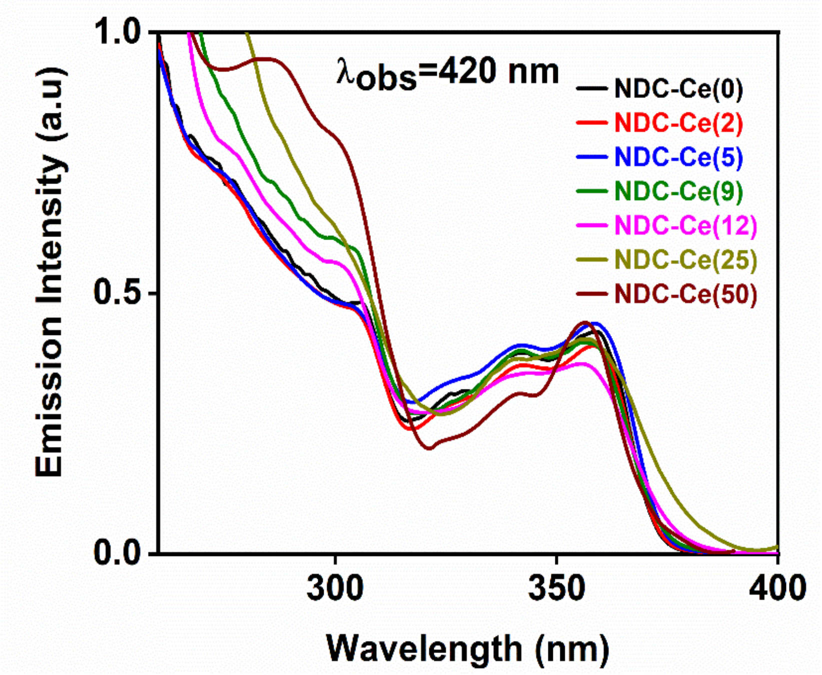

Figure S4: Excitation spectra of NDC-Ce(x) MOFs in DCM solvent. The observation wavelength was 420 nm. Due to the low emission intensity signal of NDC-Ce(75) and NDC-Ce(100), the corresponding excitation spectra could not be obtained.

8. Emission decay traces of NDC-Ce(x)s in DCM suspension.

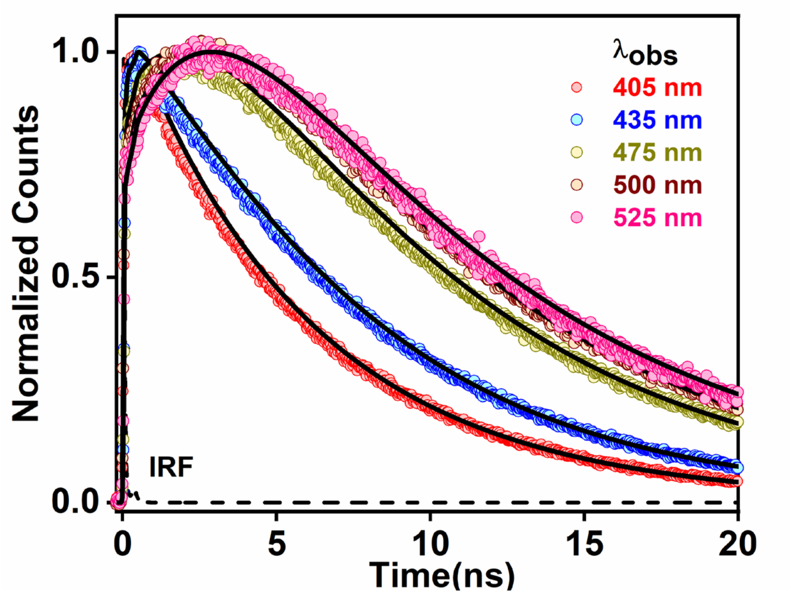

Figure S5. Magic-angle emission decays of NDC-Ce(0) in DCM solvent. The observation wavelengths are indicated in the figure and the sample was excited at 371 nm. The solid lines are from the best-fit using a multiexponential function. The IRF is the instrumental response function.

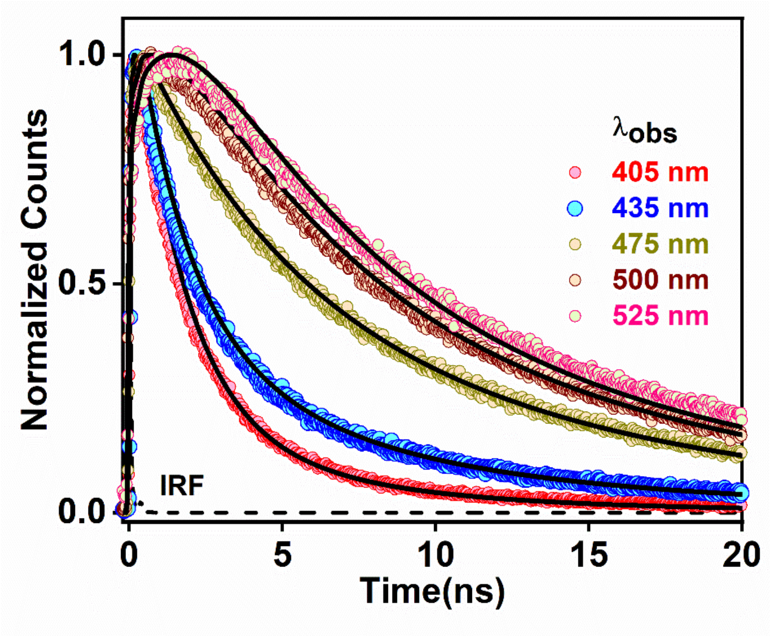

Figure S6. Magic-angle emission decays of NDC-Ce(2) in DCM solvent. The observation wavelengths are indicated in the figure and the sample was excited at 371 nm. The solid lines are from the best-fit using a multiexponential function. The IRF is the instrumental response function.

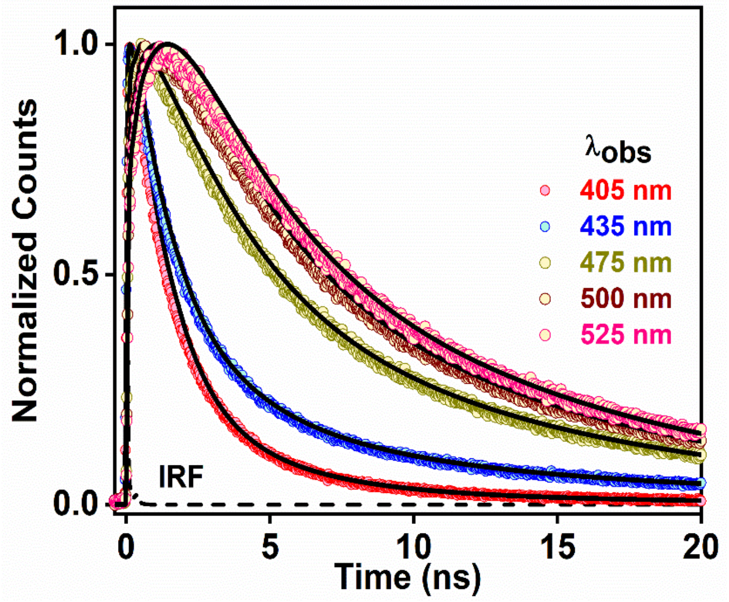

Figure S7. Magic-angle emission decays of NDC-Ce(5) in DCM solvent. The observation wavelengths are indicated in the figure and the sample was excited at 371 nm. The solid lines are from the best-fit using a multiexponential function. The IRF is the instrumental response function.

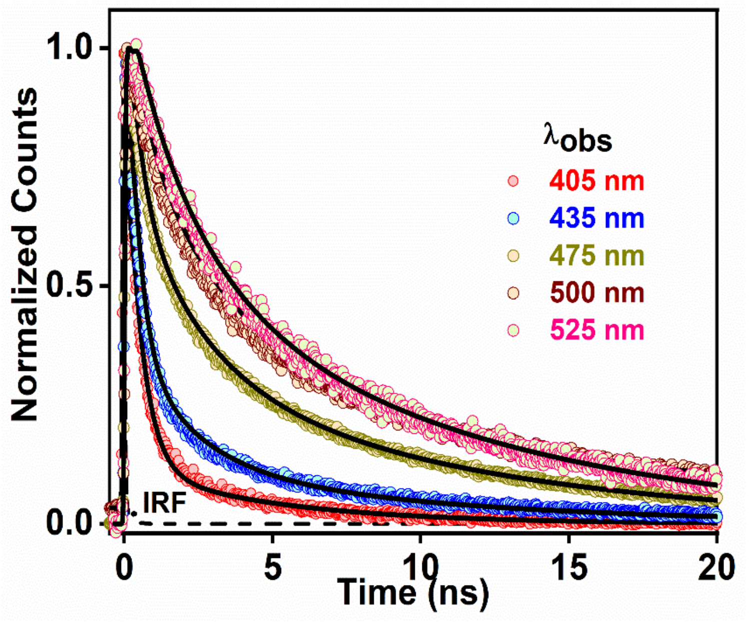

Figure S8. Magic-angle emission decays of NDC-Ce(9) in DCM solvent. The observation wavelengths are indicated in the figure and the sample was excited at 371 nm. The solid lines are from the best-fit using a multiexponential function. The IRF is the instrumental response function.

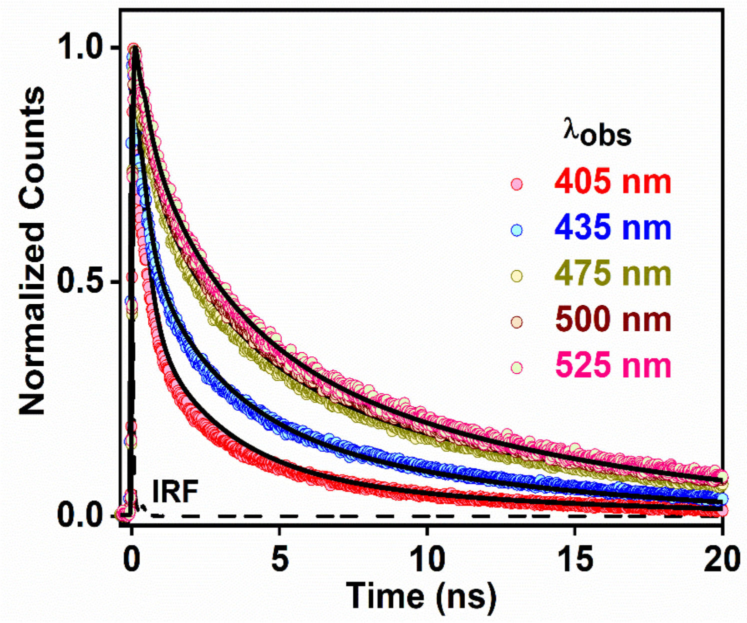

Figure S9. Magic-angle emission decays of NDC-Ce(12) in DCM solvent. The observation wavelengths are indicated in the figure and the sample was excited at 371 nm. The solid lines are from the best-fit using a multiexponential function. The IRF is the instrumental response function.

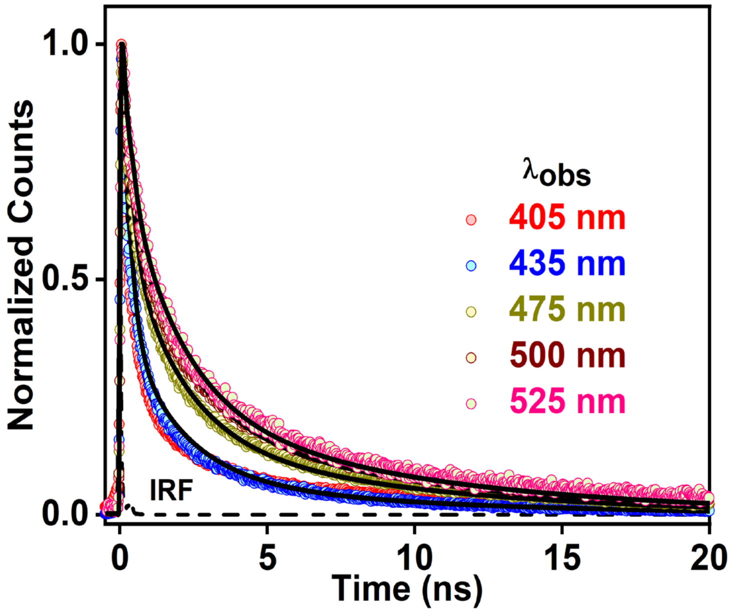

Figure S10. Magic-angle emission decays of NDC-Ce(25) in DCM solvent. The observation wavelengths are indicated in the figure and the sample was excited at 371 nm. The solid lines are from the best-fit using a multiexponential function. The IRF is the instrumental response function.

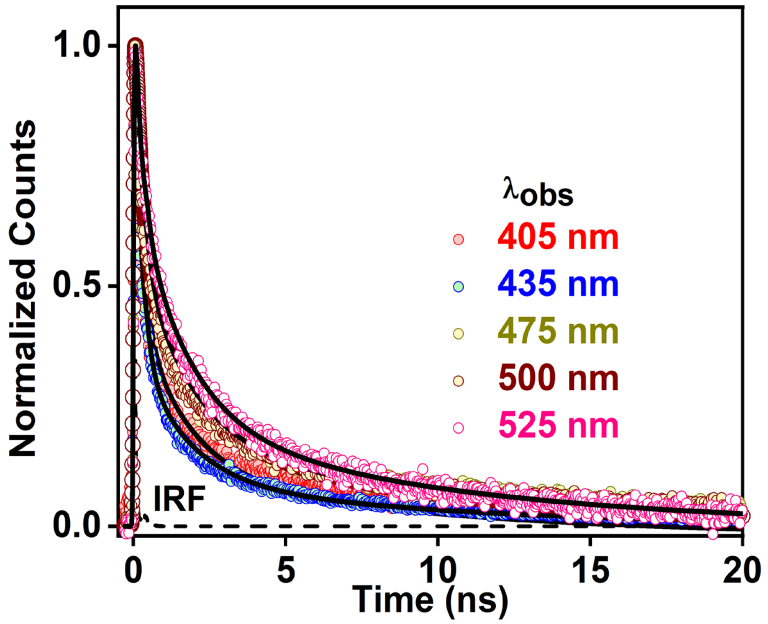

Figure S11. Magic-angle emission decays of NDC-Ce(50) in DCM solvent. The observation wavelengths are indicated in the figure and the sample was excited at 371 nm. The solid lines are from the best-fit using a multiexponential function. The IRF is the instrumental response function.

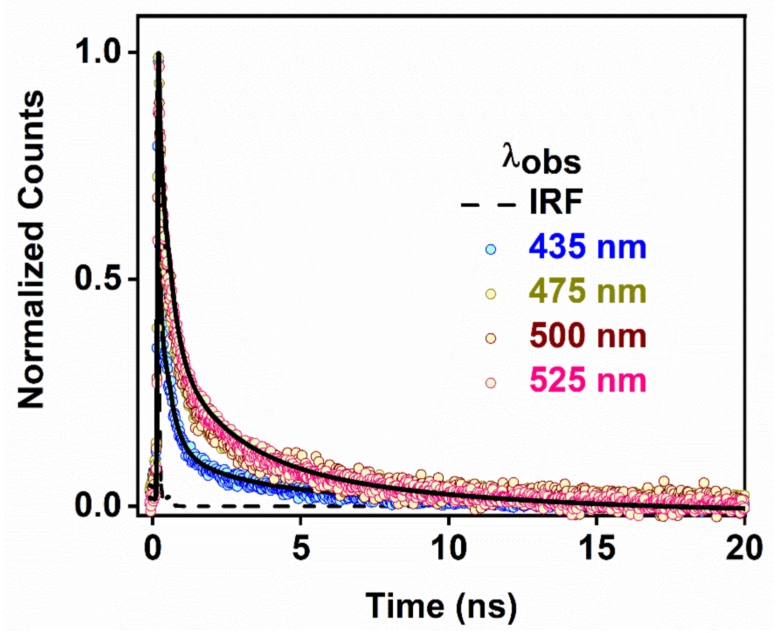

Figure S12. Magic-angle emission decays of NDC-Ce(75) in DCM solvent. The observation wavelengths are indicated in the figure and the sample was excited at 371 nm. The solid lines are from the best-fit using a multiexponential function. The IRF is the instrumental response function.

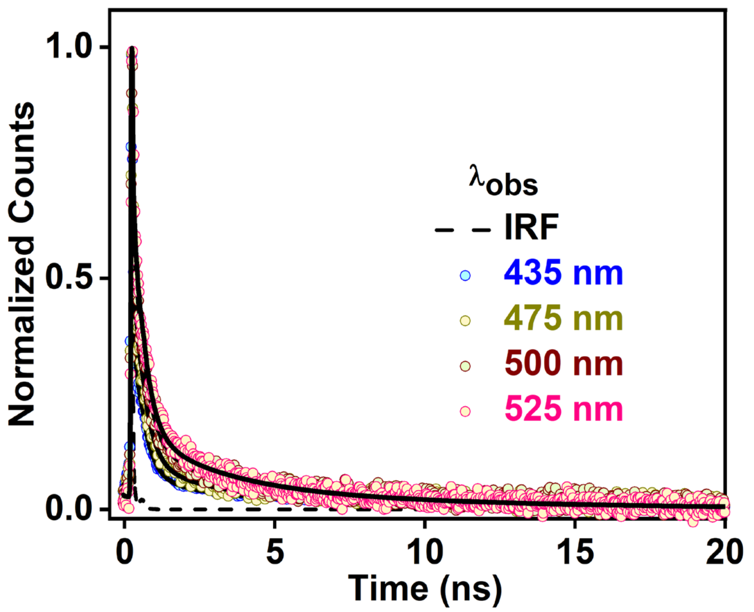

Figure S13. Magic-angle emission decays of NDC-Ce(100) in DCM solvent. The observation wavelengths are indicated in the figure and the sample was excited at 371 nm. The solid lines are from the best-fit using a multiexponential function. The IRF is the instrumental response function.

9. Tables of the parameters obtained from the fitting of the decay traces of NDC-Ce(x) MOFs in DCM suspension.

Table S4. Values of time constants (τ_i) and normalized (to 100) preexponential factors (a_i) obtained from the fit of the emission decays of NDC-Ce(0) in DCM. Negative sign of a_i indicates a rising component. The estimated uncertainty of the time constants, considering the errors from the experiments as well as those arising from the multi-exponential fit of the signals, was between 10 and 15%.

MOF	λ_{obs} (nm)	τ_1 (ns)	a_1	C_1	τ_2 (ns)	a_2	C_2	τ_3 (ns)	a_3	C_3
	**405**		7	4		90	88		3	8
	**435**		4	3		91	81		5	16
**NDC-Ce(0)**	**475**	**3.2**	(-)100	(-)100	**7.0**	73	57	**14.5**	27	43
	**500**		(-)100	(-)100		63	46		37	54
	**525**		(-)100	(-)100		58	42		42	58

Table S5. Values of time constants (τ_i) and normalized (to 100) preexponential factors (a_i) obtained from the fit of the emission decays of NDC-Ce(2) in DCM. Negative sign of a_i indicates a rising component. The estimated uncertainty of the time constants, considering the errors from the experiments as well as those arising from the multi-exponential fit of the signals, was between 10 and 15%.

MOF	λ_{obs}(nm)	τ_1 (ns)	a_1	C_1	τ_2 (ns)	a_2	C_2	τ_3 (ns)	a_3	C_3
NDC-Ce(2)	405	**2.0**	80	51	**5.1**	18	36	**13.0**	2	13
	435		58	21		32	36		10	43
	475		(-)100	(-)100		62	32		38	68
	500		(-)100	(-)100		61	30		39	70
	525		(-)100	(-)100		60	28		40	72

Table S6. Values of time constants (τ_i) and normalized (to 100) preexponential factors (a_i) obtained from the fit of the emission decays of NDC-Ce(5) in DCM. Negative sign of a_i indicates a rising component. The estimated uncertainty of the time constants, considering the errors from the experiments as well as those arising from the multi-exponential fit of the signals, was between 10 and 15%.

MOF	λ_{obs}(nm)	τ_1 (ns)	a_1	C_1	τ_2 (ns)	a_2	C_2	τ_3 (ns)	a_3	C_3
**NDC-Ce(5)**	**405**	**1.5**	72	39	**3.2**	25	41	**13.0**	3	20
	**435**		55	17		33	32		12	41
	**475**		(-)100	(-)100		60	30		40	70
	**500**		(-)100	(-)100		58	25		42	75
	**525**		(-)100	(-)100		55	20		45	80

Table S7. Values of time constants (τ_i) and normalized (to 100) preexponential factors (a_i) obtained from the fit of the emission decays of NDC-Ce(9) in DCM. The estimated uncertainty of the time constants, considering the errors from the experiments as well as those arising from the multi-exponential fit of the signals, was between 10 and 15%.

MOF	λ_{obs}(nm)	τ_1 (ns)	a_1	C_1	τ_2 (ns)	a_2	C_2	τ_3 (ns)	a_3	C_3
	**405**		82	25		14	28		4	47
	**435**		70	12		20	22		10	66
	**475**		40	6		32	21		28	73
**NDC-Ce(9)**	**500**	**0.5**	24	4	**2.5**	40	21	**11.4**	36	75
	**525**		5	1		45	17		50	82

Table S8. Values of time constants (τ_i) and normalized (to 100) preexponential factors (a_i) obtained from the fit of the emission decays of NDC-Ce(12) in DCM. The estimated uncertainty of the time constants, considering the errors from the experiments as well as those arising from the multi-exponential fit of the signals, was between 10 and 15%.

MOF	λ_{obs}(nm)	τ_1 (ns)	a_1	C_1	τ_2 (ns)	a_2	C_2	τ_3 (ns)	a_3	C_3
	**405**		66	16		27	34		7	50
	**435**		51	10		31	26		18	64
	**475**		34	6		34	20		32	74
**NDC-Ce(12)**	**500**	**0.4**	29	3	**3.0**	34	18	**12.3**	37	79
	**525**		27	2		35	18		38	80

Table S9. Values of time constants (τ_i) and normalized (to 100) preexponential factors (a_i) obtained from the fit of the emission decays of NDC-Ce(25) in DCM. The estimated uncertainty of the time constants, considering the errors from the experiments as well as those arising from the multi-exponential fit of the signals, was between 10 and 15%.

MOF	λ_{obs}(nm)	τ_1 (ns)	a_1	C_1	τ_2 (ns)	a_2	C_2	τ_3 (ns)	a_3	C_3
NDC-Ce(25)	**405**	0.3	74	17	1.8	20	38	8.4	6	45
	**435**		70	15		24	40		6	45
	**475**		54	7		33	33		13	60
	**500**		45	5		37	30		18	65
	**525**		43	4		40	30		17	66

Table S10. Values of time constants (τ_i) and normalized (to 100) preexponential factors (a_i) obtained from the fit of the emission decays of NDC-Ce(50) in DCM. The estimated uncertainty of the time constants, considering the errors from the experiments as well as those arising from the multi-exponential fit of the signals, was between 10 and 15%.

MOF	λ_{obs}(nm)	τ_1 (ns)	a_1	C_1	τ_2 (ns)	a_2	C_2	τ_3 (ns)	a_3	C_3
NDC-Ce(50)	**405**	0.3	64	8	1.6	22	30	9.5	14	62
	**435**		62	11		38	29		10	60
	**475**		58	6		28	24		14	70
	**500**		56	6		30	24		14	70
	**525**		47	4		36	24		17	72

Table S11. Values of time constants (τ_i) and normalized (to 100) preexponential factors (a_i) obtained from the fit of the emission decays of NDC-Ce(75) in DCM. The estimated uncertainty of the time constants, considering the errors from the experiments as well as those arising from the multi-exponential fit of the signals, was between 10 and 15%.

MOF	λ_{obs}(nm)	τ_1 (ns)	a_1	C_1	τ_2 (ns)	a_2	C_2	τ_3 (ns)	a_3	C_3
NDC-Ce(75)	405*	0.2	-		2.0	-		8.1	-	
	435		80	12		30	35		5	43
	475		73	10		34	32		7	58
	500		68	8		31	26		9	66
	525		64	5		30	28		11	67

*Note that due to the low emission intensity, the data at 405 nm could not be recorded.

Table S12. Values of time constants (τ_i) and normalized (to 100) preexponential factors (a_i) obtained from the fit of the emission decays of NDC-Ce(100) in DCM. The estimated uncertainty of the time constants, considering the errors from the experiments as well as those arising from the multi-exponential fit of the signals, was between 10 and 15%.

MOF	λ_{obs}(nm)	τ_1 (ns)	a_1	C_1	τ_2 (ns)	a_2	C_2	τ_3 (ns)	a_3	C_3
NDC-Ce(100)	405*	0.3	-		2.3	-		9.0	-	
	435		90	18		7	36		3	46
	475		86	20		11	35		3	45
	500		82	19		15	38		3	43
	525		80	18		15	38		5	44

*Note that due to the low emission intensity, the data at 405 nm could not be recorded.

10. μs-TA decays at two wavelengths in full temporal scale.

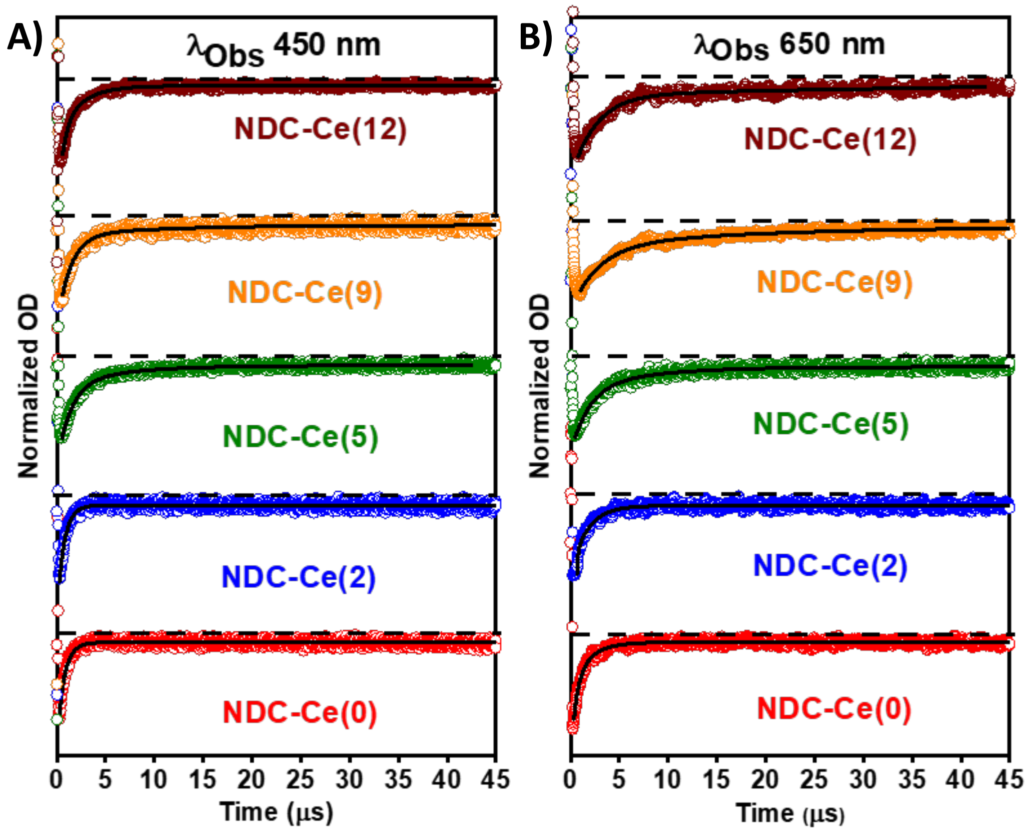

Figure S14. μs-TA decays of NDC-Ce(0-12) MOFs in DCM suspensions recorded at **A)** 450 nm and **B)** 650 nm in the full temporal scale.

11. The photo action plot of the NDC-Ce MOFs

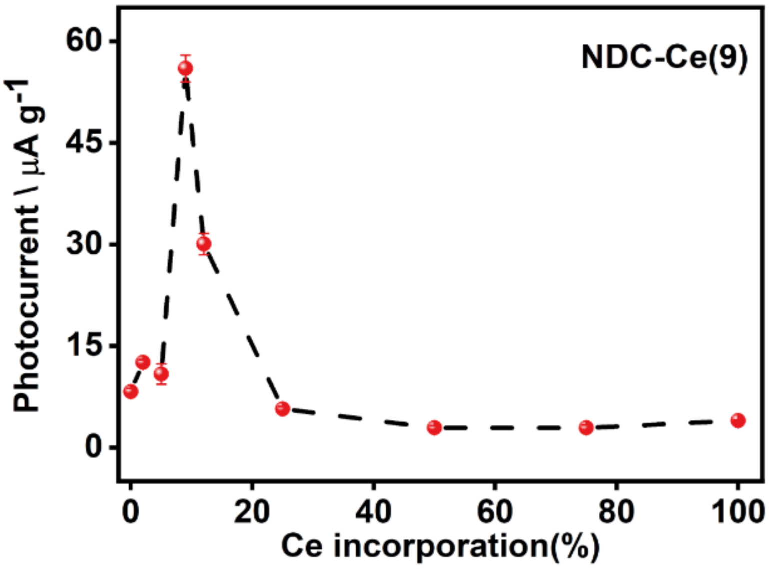

Figure S15. Plot of the maximum photo current density intensity as a function of Ce percentage of the NDC-Ce(x) MOFs.

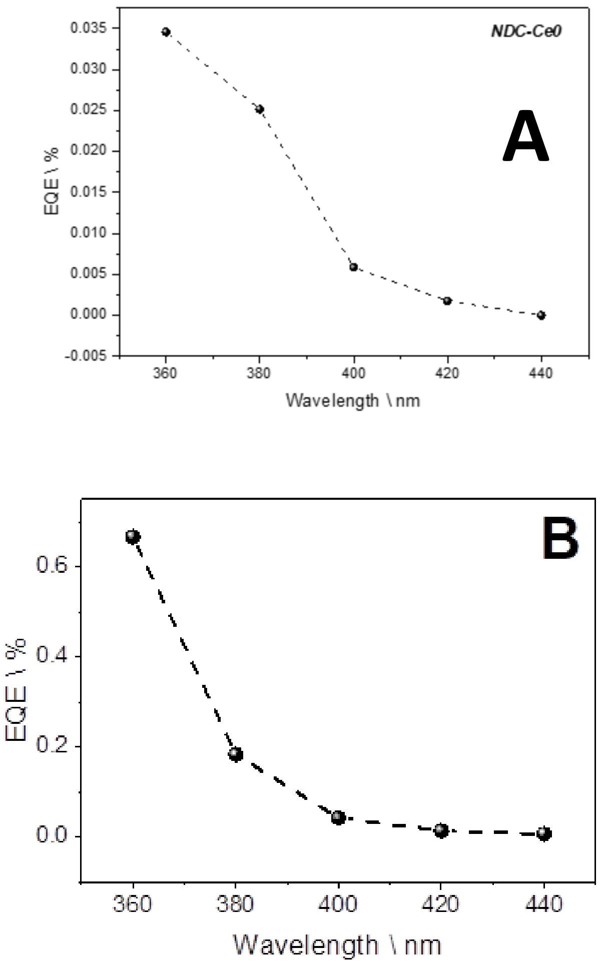

Figure S16. A) The photo action plot of NDC-Ce(0) and **B)** NDC-Ce(9), respectively, acquired with monochromatic irradiation at different wavelengths.

12. Photocurrent measurements of NDC-Ce (0) electrodes and NDC-Ce (9) using cut-off irradiation.

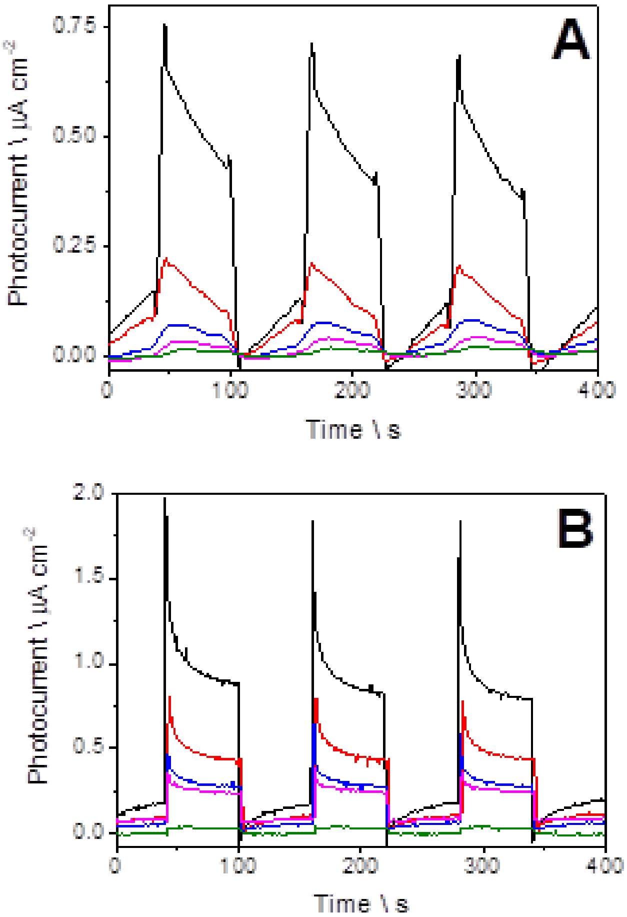

Figure S17. Photocurrent measurements of **A)** NDC-Ce(0) electrodes and **B)** NDC-Ce(9), respectively. In both plots, the black line corresponds to full Xe irradiation, red is with a 360nm cut-off filter, blue is with a 380nm cut-off filter, pink is with a 400nm cut-off filter and green is with a 450nm cut-off filter.

13. Initial H_2 evolution rates of the NDC-Ce (0) and NDC-Ce (9).

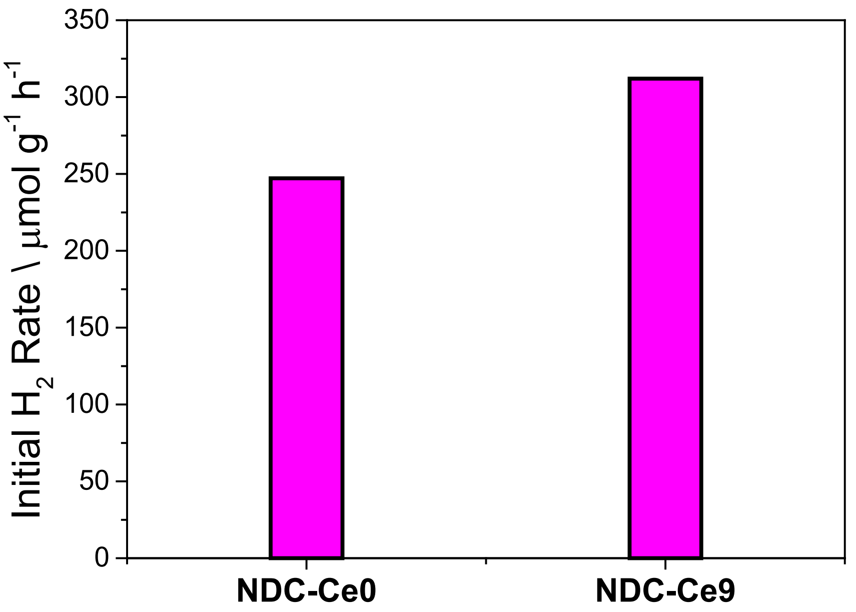

Figure S18. Initial H_2 evolution rates obtained for the NDC-Ce(0) and NDC-Ce(9).

14. Nyquist plots for NDC-Ce (0) and NDC-Ce (9)

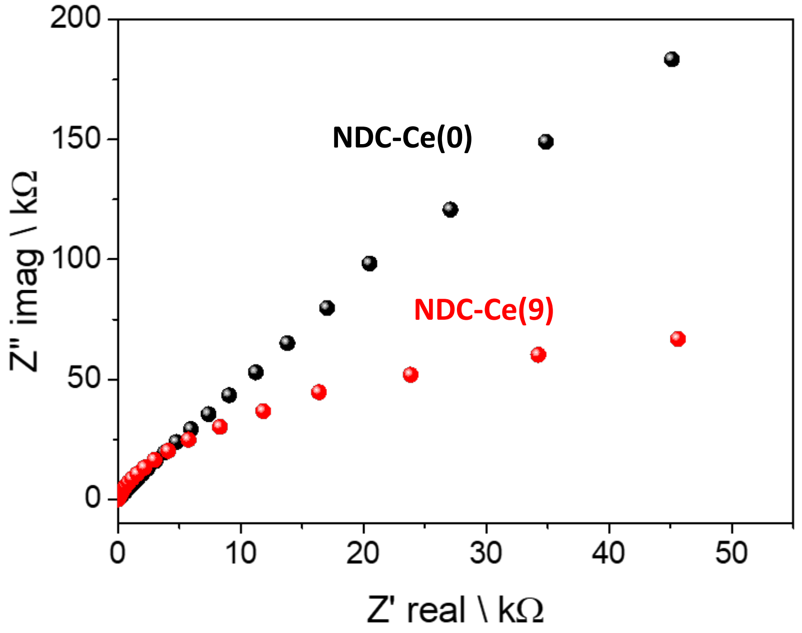

Figure S19. The Nyquist plots of NDC-Ce (0) (black) and NDC-Ce(9) (red), respectively. All data were recorded under full Xe lamp irradiation in a frequency range between 0.1 and 10^6 Hz.

15. Mott-Schottky plots for NDC-Ce (0) and NDC-Ce (9).

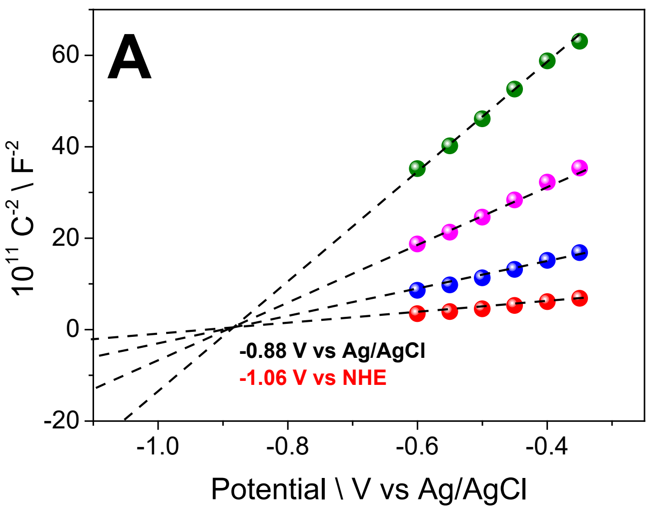

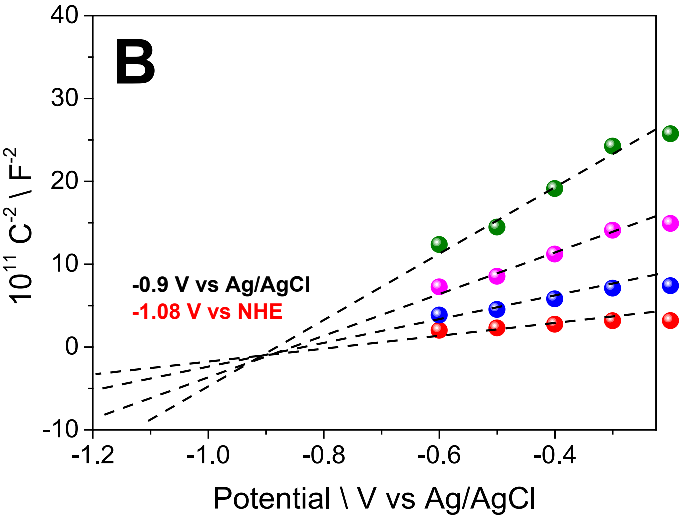

Figure S20. The Mott-Schottky plots of **A)** NDC-Ce (0) and **B)**NDC-Ce(9). All data were recorded at potentials between -1V and 1V vs. Ag/AgCl. The red dots correspond to 1500Hz, blue to 2000Hz, pink to 2500Hz and green to 3000Hz.

16. References

1. Geravand, E.; Farzaneh, F.; G.S. Millan, R.; Carmona, F. J.; Navarro, J. A. R., Mixed-Metal Cerium/Zirconium MOFs with Improved Nerve Agent Detoxification Properties. *Inorg. Chem.* **2020,** *59* (22), 16160-16167.
2. Lammert, M.; Glißmann, C.; Stock, N., Tuning the Stability of Bimetallic Ce(IV)/Zr(IV)-Based MOFs with UiO-66 and MOF-808 Structures. *Dalton Trans.* **2017,** *46*, 2425-2429.
3. Randino, C.; Ziółek, M.; Gelabert, R.; Organero, J. A.; Gil, M.; Moreno, M.; Lluch, J. M.; Douhal, A., Photo-Deactivation Pathways of a Double H-bonded Photochromic Schiff Base Investigated by Combined Theoretical Calculations and Experimental Time-Resolved Studies. *Phys. Chem. Chem. Phys.* **2011,** *13*, 14960-14972.
4. Gutiérrez, M.; Di Nunzio, M. R.; C. Mancebo, E.; Sánchez, F.; Cohen, B.; Douhal, A., Disentangling the Complex Photodynamics of Mixed-Linker Zr-MOFs – Efficient Energy and Charge Transfer Processes. *J. Mater. Chem. C.* **2023,** *11*, 183-195.
